# The evolution of *S100A7:* an unusual gene expansion in *Myotis* bats

**DOI:** 10.1186/s12862-019-1433-0

**Published:** 2019-05-14

**Authors:** Ana Águeda-Pinto, L. Filipe C. Castro, Pedro J. Esteves

**Affiliations:** 1 CIBIO/InBio, Centro de Investigação em Biodiversidade e Recursos genéticos, Universidade do Porto, Rua Padre Armando Quintas, 4485-661 Vairão, Portugal; 2 Departamento de Biologia, Faculdade de Ciências da Universidade do Porto, Rua do Campo Alegre S/N, 4169-007 Porto, Portugal; 3 CIIMAR/CIMAR, Centro Interdisciplinar de Investigação Marinha e Ambiental, Universidade do Porto, Av. general Norton de Matos S/N, 4450-208 Matosinhos, Portugal; 4 CESPU, Instituto de Investigação e Formação Avançada em Ciências e Tecnologias da Saúde, Rua Central de Gandra 1317, 4585-116 Gandra, Portugal

**Keywords:** S100A7 (psoriasin), Chiroptera, Gene duplication, Model of concerted and birth-and-death evolution

## Abstract

**Background:**

The *S100A7* gene, also called psoriasin, was first described as an upregulated protein in psoriatic skin. For the past years, the importance of this protein as a key effector of innate immunity has been clearly established, not only due to its importance protecting against bacteria skin insult in humans, but also because of its important role in amplifying inflammatory processes. Given the importance of S100A7 in host defense, *S100A7* genes have been mostly studied in humans. Here we provide a detailed analysis of the evolution of the gene family encoding for the S100A7 protein in mammals.

**Results:**

Examination of several mammalian genomes revealed an unexpected variation in the copy number of *S100A7*. Among the most representative mammalian groups, we report that multiple events of duplication, gene loss and high mutation rates are shaping the evolution of this gene family. An unexpected result comes from *Myotis* species (order Chiroptera), where we found an outstanding *S100A7* gene radiation, resulting in more than 10 copies in *M. lucifugus* and 5 copies in *M. brandtii*. These findings suggest a unique adaptive road in these species and are suggestive of special role of this protein in their immune system.

**Conclusions:**

We found different evolutionary histories among different mammalian groups. Overall, our results suggest that this gene family is evolving under the birth-and-death model of evolution. To our knowledge, this work represents the first detailed analysis of phylogenetic relationships of *S100A7* within mammals and therefore will pave the way to further clarify their unique function in the immune system.

**Electronic supplementary material:**

The online version of this article (10.1186/s12862-019-1433-0) contains supplementary material, which is available to authorized users.

## Background

S100A7 (S100 Calcium Binding Protein A7) is a member of the S100 protein family, sharing the typical EF-hand helix-loop-helix domain that is responsible for the calcium binding function [1]. S100A7 was first identified as an upregulated protein in psoriatic keratinocytes, having an important role in hyperplasia and in inflammatory processes observed in psoriatic skin lesions [2]. S100A7 is not only overexpressed in psoriatic skin, but also in atopic dermatitis, Darier disease, mycosis fungoides and skin cancer, suggesting a major role for this protein in inflammation and keratinocyte differentiation [3–7]. Several studies show that high levels of *S100A7* expression occur in response to several cytokines, including IL-17, IL-22 and TNF (Tumor Necrosis Factor) [8–10]. Following *S100A7* stimulation, this protein can activate different cellular signaling pathways, both in keratinocytes and neutrophils, such as AP-1 (activator protein 1), NF-κB (nuclear factor kappa-light-chain-enhancer of activated B cells), and STAT3 (signal transducer and activator of transcription 3), resulting in the upregulation of multiple pro-inflammatory cytokines and in an amplifying inflammatory process [11, 12]. Moreover, it has been established that after secretion by keratinocytes, S100A7 proteins also work as a chemotactic agent towards neutrophils and T-cells [13, 14]. Besides its chemotaxis properties, S100A7 is also an important *Escherichia coli*-cidal antimicrobial protein. *S100A7* is expressed in areas with high bacterial colonization, being one of the main antimicrobial proteins expressed in normal skin [15]. Moreover, it is the main *Escherichia coli*-killer compound, explaining why *E. coli* rarely infects healthy skin, even in areas where its concentration is high [16, 17]. Given its antimicrobial properties, along with its ability to enhance the host defense functions at sites of infection and inflammation, S100A7 has been considered a key effector of innate immunity.

Genomic *loci* analyses showed that most *S100A* genes are clustered on a single chromosome with a conserved arrangement among eutherians (e.g. human and mouse). In humans, *S100A7* genes are located in the epidermal differentiation complex (EDC) on chromosome 1q21 that comprises several other genes predominantly expressed in the skin [18, 19]. So far, the largest number of *S100A7* copies was described in primates, with *Homo sapiens* having three functional genes and two nonfunctional [20].

**Fig. 1:**
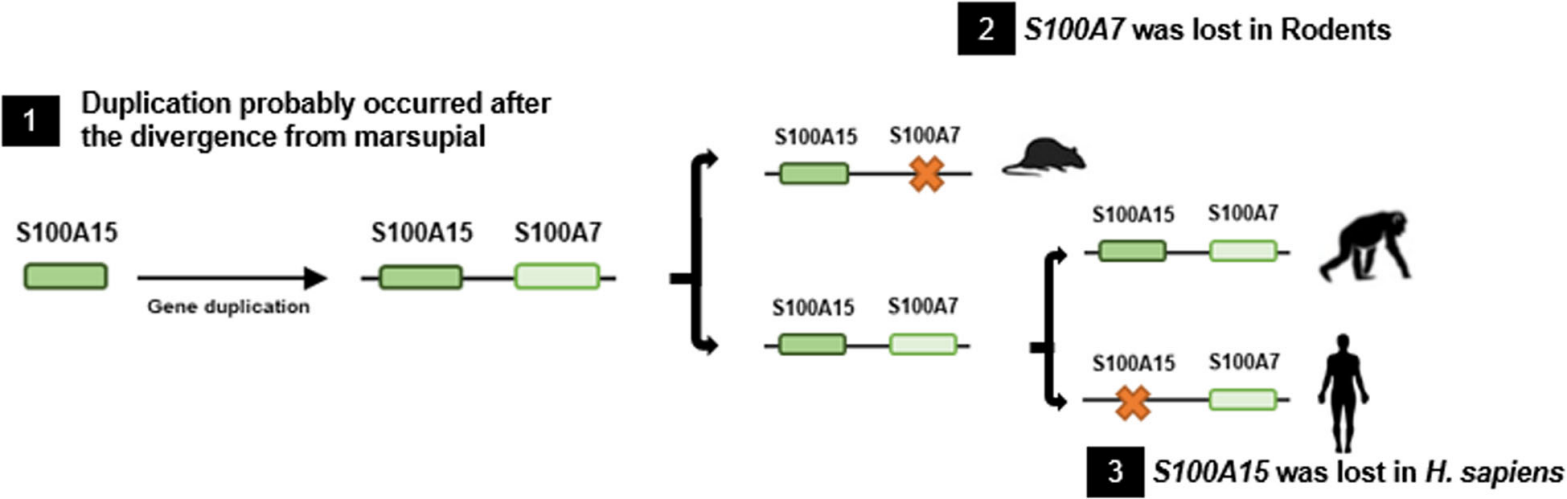
Evolutionary history of the *S100A15* and *S100A7* genes. An ancient *S100A15* gene experienced a duplication event (indicated by (1)) that gave rise to two different genes: *S100A15* and *S100A7*

Several studies support that extant eutherian species have two genes, *S100A7* and *S100A15*, likely to have originated via gene duplication after the split from marsupials around 150 million years ago (Mya) (Fig. 1) [21]. In some eutherian mammals, like human and chimpanzee, *S100A7* experienced several events of duplication, while in rodents this gene lineage was lost [22, 23]. Interestingly, the ancient *S100A15* is preserved in rodents, while was lost in the human lineage [23, 24]. Overall, *S100A7* has most likely undergone multiple events of gene rearrangement and duplication during mammalian evolution. The variable number of *S100A7* copies found in Primates along with the importance of this protein in the innate immune system, prompted us to investigate its molecular evolution among eutherian mammals. Moreover, a comparative and phylogenetic approach involving a wider range of eutherian mammals should shed some light on the evolution of *S100A7* gene family. In this study, we show that after the radiation of *S100A7* from marsupials, this gene experienced a dynamic pattern of gene duplication and loss. Surprisingly, in the *Myotis* family (Chiroptera), we show strong evidences of multiple events of duplication, followed by events of recombination and acceleration of point mutations. On the basis of the obtained results, we propose a birth-and-death model of evolution acting in this gene family.

## Results

### The *S100A7* repertoire in mammals

In this study, our main goal was to explore the evolutionary patterns of *S100A7* genes in mammals and the evolutionary forces that are shaping this gene family. For this, human *S100A7* and *S100A7A* were used to perform blastn searches and collect *S100A7-like* sequences from several available genomes. Our database search detected the presence of *S100A7* genes in seven eutherian orders, including order Artiodactyla (12 species), order Carnivora (four species), order Chiroptera (7 species), order Primates (12 species), order Cetacea (one species), order Perissodactyla (two species) and superorder Afrotheria (two species). In total, 82 sequences from 40 species were included in this study (Table 1 and Additional file 1).

**Table 1:** Number of *S100A7* copies found in different mammalian orders

Order	Family	Species name	*S100A7*
Primates	Hominidae	*Homo sapiens*	5^a^
		*Pan troglodytes*	2
		*Pan paniscus*	2
		*Gorilla gorilla*	2
	Hylobatidae	*Nomascus leucogenys*	2
	Cercopithecidae	*Papio anubis*	1
		*Macaca mulatta*	1
		*M. fascicularis*	2
		*M. nemestrina*	1
		*Rhinopithecus bieti*	2
	Cebidae	*Cebus capucinus*	1
		*Saimiri boliviensis*	1
Chiroptera	Vespertilionidae	*Myotis davidii*	3
		*Myotis brandtii*	5
		*Myotis lucifugus*	13
		*Eptesicus fuscus*	1
	Miniopteridae	*Miniopterus natalensis*	2
	Pteropodidae	*Pteropus vampyrus*	1
		*Pteropus alecto*	2
		*Rousettus aegyptiacus*	3
Perissodactyla	Equidae	*Equus caballus*	1
		*Equus asinus*	1
Artiodactyla	Bovidae	*Bos taurus*	3
		*B. indicus*	3
		*Bison bison*	2
		*Bubalus bubalis*	3
		*Pantholops hodgsonii*	2
		*Capra hircus*	3
		*Ovies aries*	2
	Suidae	*Sus scrofa*	2
	Balaenopteridae	*Balaenoptera acutorostrata*	3^b^
	Camelidae	*Camelus dromedarius*	1
		*Camelus ferus*	1
		*Camelus bactrianus*	1
		*Vicugna pacos*	1
Carnivora	Ursidae	*Ailuropoda melanoleuca*	1
		*Ursus arctos*	1
		*Ursus maritimus*	1
	Otariidae	*Callorhinus ursinus*	1
Afrotheria	Orycteropodidae	*Orycteropus afer afer*	2
	Elephantidae	*Loxodonta africana*	1^c^

^a^Presence of two described pseudogenes

^b^Presence of two partial sequences

^c^Partial sequence

Besides the complete *S100A7* coding sequences, a detailed sequence analysis suggested a number of inconsistent annotations in the NCBI and Ensembl databases. For example, we found a *S100A7A* (XM_006501635.3) and a *S100A7* annotation (AY582964.1) in mouse with 100% identity between them, suggesting that these sequences are in fact the same gene. Moreover, we observed that *S100A7* from mouse presented a low degree of similarity when aligned with the *S100A7* coding sequences from other mammals (Additional file 3). In fact, after further analysis, we found that the annotated *S100A7* from mouse was in fact a *S100A15* gene. This situation was also found in the brown rat (*Rattus norvegicus*, NM_001109471.1). Given the high degree of similarity of these genes with the *S100A15* gene from other mammals (Additional file 4), here we assume that these sequences correspond to a *S10015* gene. Three annotated *S100A7* for the minke whale (*Balaenoptera acutorostrata*, XM_007178590.1, XM_007178589.1 and XM_007198423.1) were found. The second and the third correspond to partial sequences and, for this reason, were not included in the phylogenetic analysis. Moreover, the search in the elephant (*Loxodonta africana*) genome also resulted in a partial sequence (XM_007948587.1) and was also not used in this study.

It was previously reported that *S100A7* from mouse and rat may have been lost after their separation marsupials [22, 23]. In fact, and as mentioned before, our results support these findings since no functional *S100A7* sequences were found in rodents (mouse and rat). Moreover, after a careful analysis of the mouse genome, we found remnants of the *S100A7* gene (Additional file 5) at the *S100* locus, supporting further the gene loss hypothesis. The same was also true in human genome, where we also found a partial sequence of *S100A15* gene. Moreover, the partial sequences found in mouse and human are in the same locus as the remaining *S100* genes (Additional file 5). Our database research also revealed that species like *Canis lupus* (family Canidae), *Acinonyx jubatus* and *Panthera pardus* (both from family Felidae) presented no *S100A7* annotation. However, when we further investigated the genomic region corresponding to the *S100A7* locus [22] for several canids and felines species we found that *S100A15* gene is still present (Additional file 4).

On the other hand, we found several events of *S100A7* duplication in other eutherian species (Table 1 and Additional file 1). Interestingly, blast searches at Chiroptera genomes hinted at a high number of *S100A7* genes, with six *S100A7* genes being assigned into the Pteropodidae family (suborder Megachiroptera) while the remaining sequences were assigned to the Vespertilionidae family (suborder Microchiroptera). Interestingly, the Little Brown Bat (*Myotis lucifugus*), a representative of the Vespertilionidae family (genera *Myotis*), presented 13 copies of *S100A7* gene, while *M. brandtii presented five copies,* suggesting that a rapid evolution of *S100A7* is occurring in these species. Besides the complete *S100A7* sequences, six other incomplete sequences were identified for the Chiroptera order (see Additional file 6). All the incomplete sequences belong to suborder Microchiroptera, being distributed by Megadermatidae, Rhinolophidae, Mormoopidae, Hipposideridae and Phyllostomidae families. However, these incomplete sequences were not used in the recombination and phylogenetic analyses performed in this study.

### Phylogenetic reconstruction suggests specific *S100A7* expansions in mammals

**Fig. 2:**
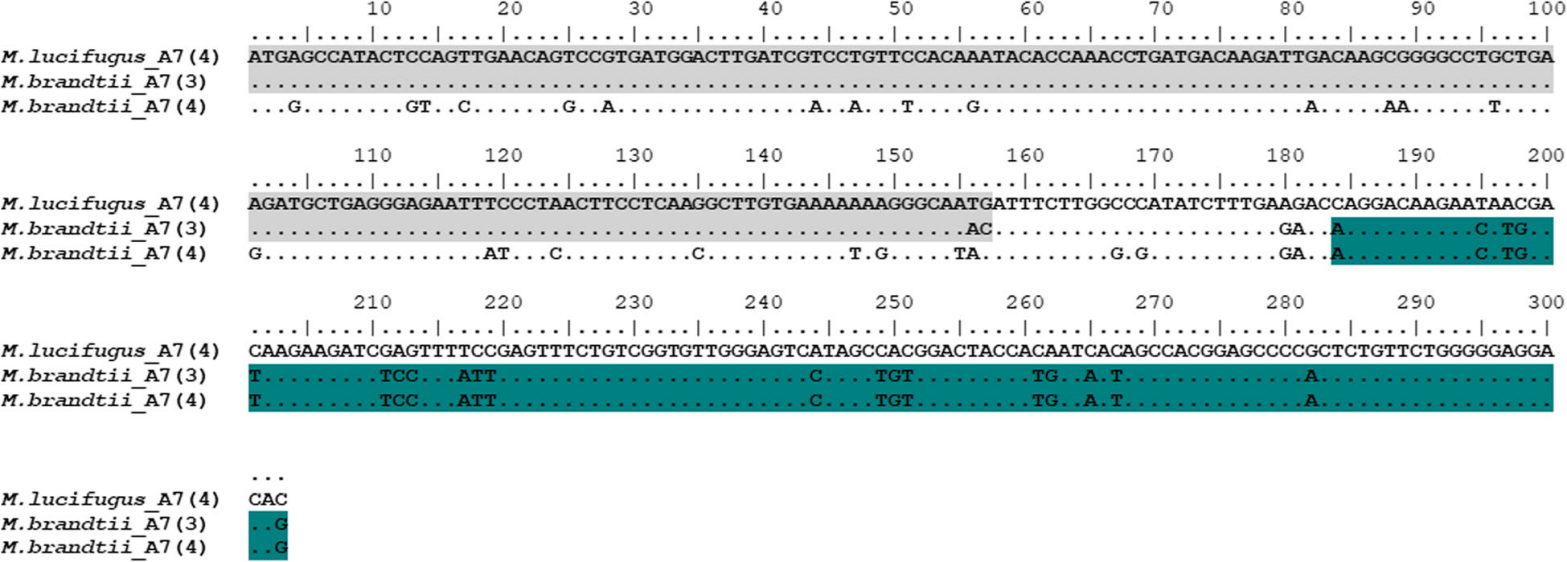
Nucleotide alignment of the recombinant *S100A7* sequence *M. brandtii*_A7(3) and the parental sequences as defined by RDP (*M. lucifugus*_A7(4) and *M. brandtii*_A7(4)). Nucleotide positions 158–183 shows the recombination breakpoint found by five different methods (*p*-values < 0.05). The grey region highlights the recombination area between *M. lucifugus*_A7(4) and *M. brandtii*_A7(3) and the blue region highlights the recombination area between *M. brandtii*_A7(4) and *M. brandtii*_A7(3)

Before further phylogenetic analyses, the retrieved sequences were screened using the RDP [25] to look for any evidence of recombination in the alignment. For the 82 complete sequences of *S100A7*, only *M. brandtii_A7(3)* presented a consistent recombination breakpoint (nucleotide positions 158–183) with strong statistical support for 5 different methods (*p*-values < 0.05) (Table 2). *M. lucifigus_A7(4)* and *M. brandtii_A7(4)* were identified as the donors of this sequence (Table 2). From the obtained results, it seems that the recombination event involved an ancestral *S100A7* gene of *M. brandtii*, very similar to the sequence of *M. lucifigus_A7(4)*, which in the process of evolution might have been lost (Fig. 2). Nevertheless, we cannot rule out the hypothesis that this gene can be present in the genome of *M. brandtii*, but it is not available in public databases. Taking into consideration that recombination events might interfere with the phylogenetic relationships of *S100A7 s*equences, *M. brandtii_A7(3)* was not included in the phylogenetic analysis.

**Table 2:** Results of the recombination analysis of the *S100A7* genes using RDP

	Most likely donor sequence		Breakpoint	Methods (average *P*-value)					
Sequence	Major Parent	Minor Parent	Start	GENECONV	BootScan	MaxChi	Chimaera	SIScan	3Seq
*M. brandtii_A7(3)*	*M. lucifugus_A7(4)*	*M. brandtii_A7(4)*	158–183	2.53 × 10^− 02^	1.05 × 10^− 02^	6.05 × 10^− 07^	1.05 × 10^−07^	1.147 × 10^−06^	–

**Fig. 3:**
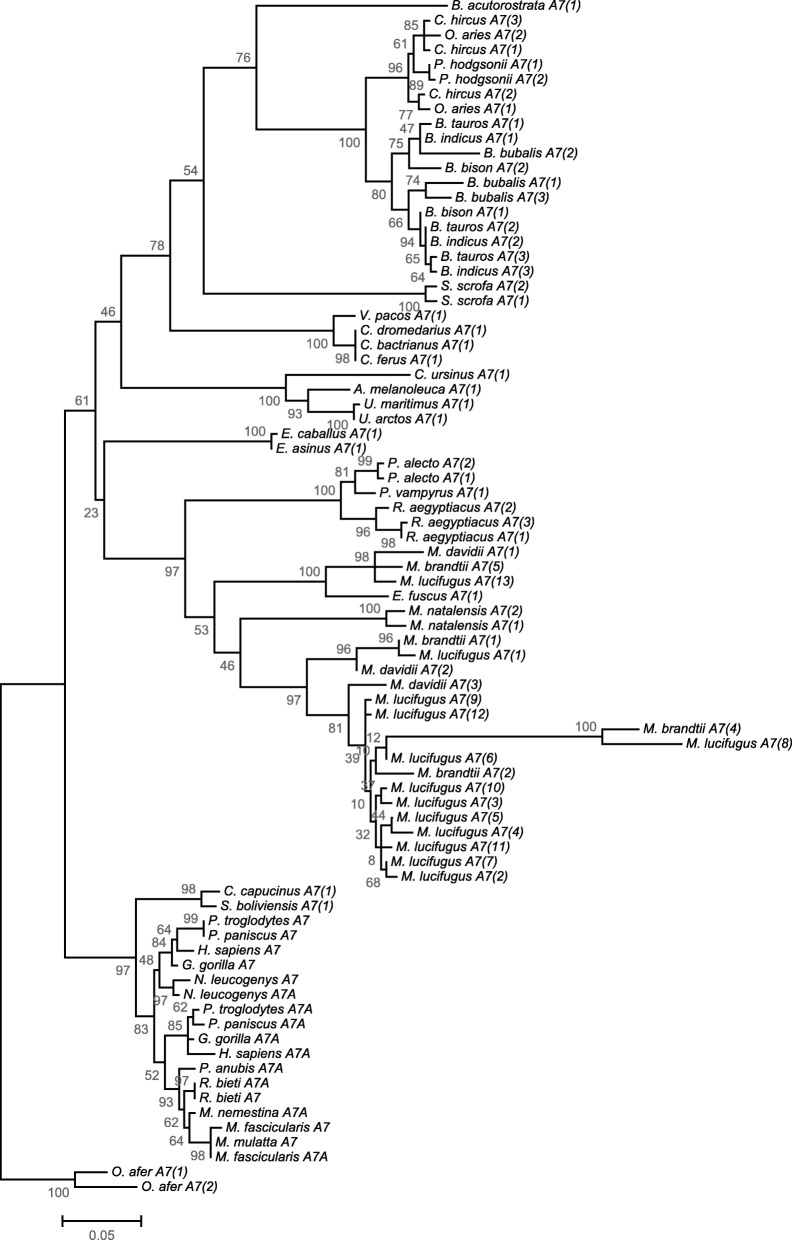
Phylogenetic analysis of *S100A7* genes in eutherian mammals. The analysis were performed with 1000 generations and 1000 bootstrap searches. Bootstrap values (%) are indicated on the branches. The abbreviations correspond to the ones shown in Additional file 1

In the *S100A7* phylogenetic reconstruction, a total of seven mammalian orders are represented. It can be observed a concordant topology with the accepted evolutionary relationships of the eutherian mammals [26, 27]. However, the monophyly of these groups is not statistically supported by the bootstraps analysis (Fig. 3). Given the topology of the phylogenetic tree, it seems that after the radiation of the *S100A7* ancestor gene it continued to be subject to gene duplication and loss. We have previously shown that *S100A7* gene family has been shaped by multiple events of duplication over ~ 35 Mya of primate evolution [28], predating the evolutionary origins of the divergence of Platyrrhini and Catarrhini primates. In Fig. 3, it is clear that these duplication events are not specific to Primate lineage. For example, regarding the order Chiroptera, it is possible to observe that in the Pteropodidae family, *P. vampyrus* maintained a single copy of *S100A7*, while in *P. alecto* and *R. aegyptiacus* the single copy ancestor of *S100A7* experienced duplication, resulting in a total of two and three genes, respectively. In Vespertilionidae, our ML tree further suggests that a radiation of *S100A7* occurred before the divergence of early *Myotis* species (around 20 Mya [29–31]). Strikingly, in *M. lucifugus*, at least 10 copies can be found, which suggest the occurrence of multiple duplication events. Accordingly, duplication events can also be observed in family Bovidae (Fig. 3).

**Fig. 4:**
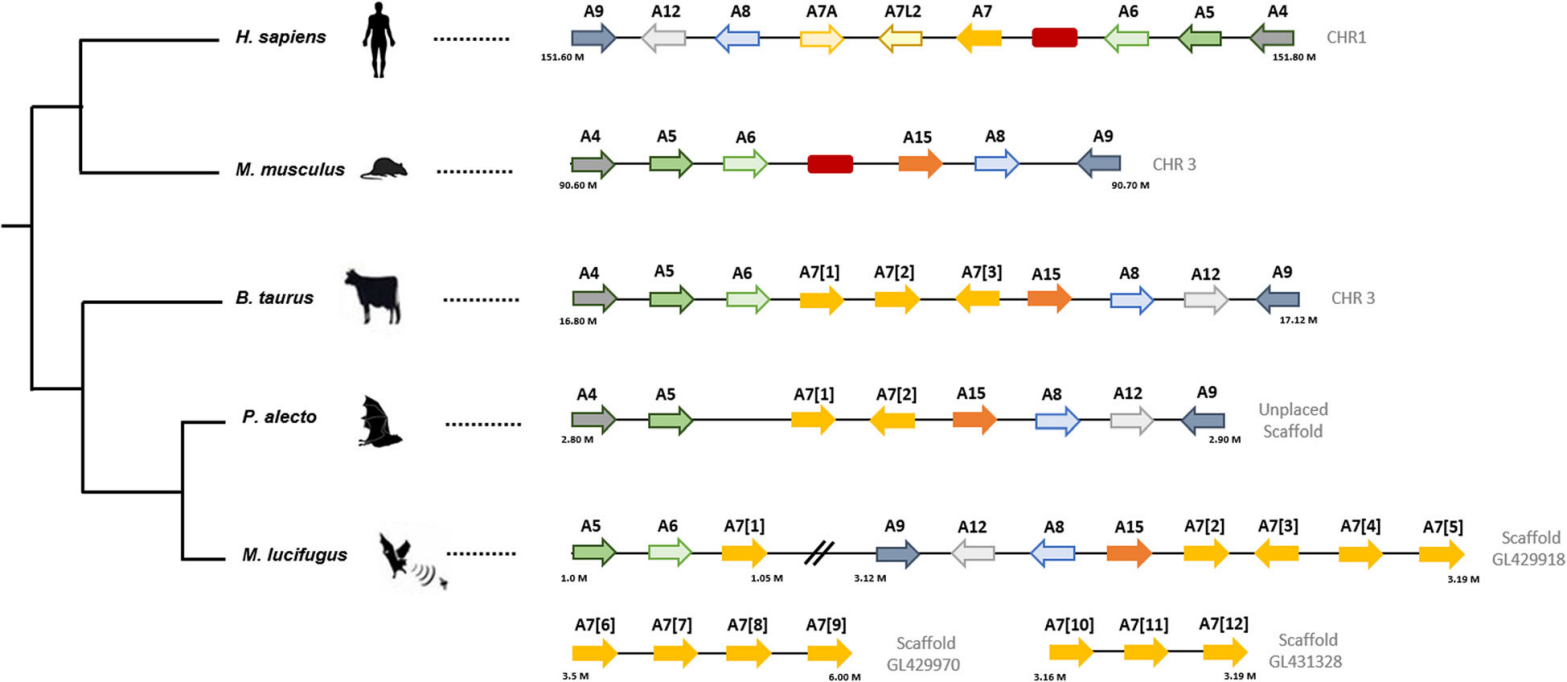
Comparison of *S100A* gene loci on human, mouse, domestic cow, flying fox and microbat. Comparison between these species show good conservation of most genes in this region and highlights that *S100A7* experienced multiple duplications in different species. Each *S100A* gene is represented in a different color. Putative pseudogenes are represented by red boxes. Orientation of the genes are represented by an arrow

Genomic *loci* analysis clearly showed that the *S100A* genes clustered in close proximity in a single chromosome in mammals (human, mouse and opossum): the *S100A3*, *S100A4*, *S100A5* and *S100A6* form a single cluster, next to *S100A7, S1000A15, S100A8*, *S100A12* and *S100A9* genes [32, 33]. Thus, we next analyzed the genomic organization of *S100A7* genes and respecting flanking genes from five different species: human, mouse, domestic cow (*B. taurus*), flying fox (*P. alecto*) and microbat (*M. lucifugus*) (Fig. 4). The analysis of the flanking genes revealed that in each of these five mammals, *S100A7* multi-copies are clustered between *S100A9*, *S100A12* and *S100A8* and *S100A15, S100A6* and *S100A5*, suggesting a conserved gene rearrangement between these species. However, it should be notice that these gene clusters are in opposite orientation from the human *S100A7* gene locus (Fig. 4). Regarding *M. lucifugus*’ *S100A7* copies, we found that these genes are dispersed for three different scaffolds: gl429918, gl429970 and gl431328. Among these genes, five are in close proximity with the expected two clusters in the scaffold gl429918, following the same arrangement as the *S100A7* copies of the other mammalian species (Fig. 4). Since the scaffold gl429918 ends right after the position of the *M. lucifugus_A7*(5) gene, it is possible that the position of the remaining genes in two other scaffolds do not allow us to infer the true location of these genes in the genome. Overall, the species that presented multiple copies of the *S100A7* gene revealed a conserved synteny among them and, therefore, further support the hypothesis that multiple events of duplication are shaping the evolution of this gene.

### Evidence of a high mutation rate in specific *S100A7* gene lineages

From the obtained ML tree, and taking in consideration the longer branch lengths, it appears that *M. lucifugus_A7(8)* and *M. brandtii_A7(4)* might be experiencing an acceleration of the mutation rate, compared to the remaining *S100A7* sequences (Fig. 3). In the Tajima’s test, the hypothesis that the compared genes have equal rates of mutation was rejected (Table 3). In fact, from the Tajima’s test results it is possible to observe that *M. lucifugus_A7(8)* and *M. brandtii_A7(4)* are experiencing a rate of mutation two times higher than the other *S100A7* genes.

**Table 3:** Results of Tajima’s relative rate test

Sequence A	Sequence B	Outgroup	Identical sites	Divergent sites	Sequence A Specific	Sequence B Specific	Outgroup Specific	X^2^ test	*p*-value^1^
*M. lucifugus_A7(8)*	*M. lucifugus_A7(13)*	*E. caballus_A7*(1)	196	6	49	24	28	8.56	0.003
*M. brandtii_A7(4)*	*M. brandtii_A7(5)*	*E. asinus_A7*(1)	196	6	47	27	27	5.41	0.020

^1^P-value <0.05 was used to reject the hypothesis of equal rates between the three lineages

**Fig. 5:**
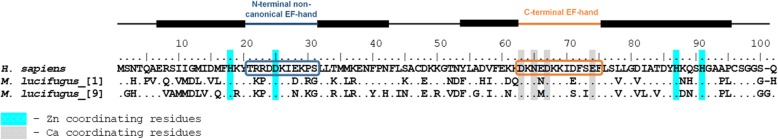
*S100A7* sequences from different species highlighting the calcium- and zinc-binding residues and the EF-hand domains

Given the high mutation rates observed, selective pressures acting on this branch were calculated using codeml of the PAML 4.9 package [34]. Codeml results suggested an intensification of selective pressures, when compared to the remaining branches, with an estimation of ω = 1.44, indicating that this branch is under positive selection (Additional file 7). Moreover, RELAX analysis also confirmed an intensification of the mutation rates (Additional file 7). Considering the S100A7 protein structure, it is interesting to observe that despite *M. lucifugus_A7* [8] presents around 32 and 44% of amino acid differences from *M. lucifugus_A7* [4] and *H. sapiens_A7*, respectively, none of these amino acids fall within the Ca^2+^ and Zn^2+^ coordinating residues [35] (Fig. 5), suggesting a maintenance of the core function of this protein. However, although no ORF disrupting mutations were found in the coding sequences of these genes, we can not rule out the hypothesis that the accumulation of several non-synonymous mutations might indicate a pseudogenization process acting on *M. lucifugus_A7* [8] *and M. brandtii_A7* [4].

## Discussion

In the past years, *S100A7* has emerged as a key regulatory component in the immune system, not only because of its ability to defend host from invading pathogens, but also for having an important role in inflammation and keratinocyte differentiation [2, 14, 15]. So far, humans were the species with more *S100A7* genes described to date, presenting three functional copies and two nonfunctional ones [19, 20]. Although this gene family has been the focus of several studies, their evolutionary history among mammals has not been studied before. In this study, we present an extensive analysis, combining genome analysis, phylogenetics and comparative genomics to provide the complete history of the evolution of this gene.

### Dynamic evolution of the *S100A7* gene in mammals

From our results, the *S100A7* repertoire varies significantly among mammals (Fig. 3). Studies suggest that after the split from marsupial, the *S100A15* underwent a duplication event, resulting in a *S100A7* ancestor gene present only in the eutherian mammals [22]. Accordingly, we did not find any evidence of *S100A7* in marsupial species. Moreover, we found that rodents and some Carnivora lineages also lack *S100A7*. The most parsimonious explanation for the obtained data is that during evolution, these lineages lost the *S100A7* gene. Interestingly, they retain the *S100A15* gene orthologue (Additional file 4 and [22]). It has been proposed that the mouse *S100A15* parallels the structure, gene expression and protein processing patterns that the S100A7 protein from humans [23]. In fact, the loss of this protein in the mentioned mammals might suggest a compensatory effect of S100A15. However, further studies are needed to validate this theory.

### Expansion of *S100A7* in bats

Although bats represent one of the largest and diverse group of mammals and are prone to various emerging infectious diseases, little is still known about their immune system [36–38]. Vespertilionidae is the largest family in Chiroptera, with more than 400 known species [39]. However, the high number of *S100A7* genes found in this family appears to be the result of several duplicated genes in three species: *M. brandtii, M. lucifugus* and *M. davidii*, with *S100A7* copy numbers ranging from three to 13. In bats, a comparable polymorphism to that observed for *S100A7* comes from the major histocompatibility complex (MHC) class II DRB genes, which are known to play a major role in immune system [40, 41]. Indeed, a wide range of variability among different species of bats was also found in MHC loci, where up to 10 DRB loci were found in sac-winged bat (*Saccopteryx bilineata*) while in genus *Noctilio* only one locus was found [42]*.* Moreover, a study focusing in bitter taste receptor genes (*Tas2rs*) showed a lineage-specific duplication of several of these receptors that only occurred in *Myotis* bats, with the new copies of *Tas2rs* presenting functional differentiations and functional innovations following duplication [43]. It is known that bats have a high propensity to tolerate massive diseases originated by a high array of pathogens [37, 44–47]. For example, bats harbor a higher proportion of zoonotic viruses than all other mammalian orders [48, 49]. A recent study using the Egyptian Rousette (*Rousettus aegyptiacus*) shows that enhanced tolerance to infection might be a result of an expansion and diversification of several loci, as well as unique adaptations in type I interferons responses and natural killer cell signaling pathways [37].

Besides the multiple events of duplication that occurred in *S100A7* gene family in Chiroptera order, our results also show strong evidences of recombination and high rates of mutation. In fact, the phylogenetic tree presented longer branches in *M. lucifugus_A7(8)* and *M. brandtii_A7(4)* coding sequences when compared with the remaining *S100A7* genes. From our results, we observed that these genes are experiencing a high mutation rate, comparatively to the remaining *S100A7* from bats (Tables 2 and 3). Similar to our results, a previous study also reported evidences of episodes of positive selection acting on the Toll-like receptor 8 (TLR8), shaping its’ diversity throughout bats evolution [50]. Interestingly, the authors showed that positive selection is especially strong in codons located in the ligand-binding ectodomain, which might contribute to a variation in the ability of different bats to recognize molecular patterns of virus [50]. In S100 proteins, upon Ca^2+^ binding, the EF-hand motif undergoes a conformational change. This change in conformation is responsible for the exposure of a hydrophobic surface that is fundamental for target recognition [1, 35]. The same way, it is known that the protective role of S100A7 against *E. coli* is highly dependent of the binding of Zn^2+^ [16]. However, it is interesting to note that despite the high mutation rates observed in these proteins, their Ca^2+^ and Zn^2+^ motifs are not modified (Fig. 5). From the alignment of these genes, both copies appear to be functional as no obvious deleterious mutations or early stop codons were found. However, since no expression patterns are currently available for these genes, we hypothesise that a recent pseudogenization event might have affected the promoter or other regulatory region (UTR), resulting in the high mutation rates observed.

Given the wide geographic distribution of *Myotis* (except Antarctica) and the importance of S100A7 in the immune system, we suggest that these duplication events might have an important role in protecting these species when exploiting new environmental settings.

### Birth-and-death evolution model

Our results raised the puzzling question on how and why this complex evolutionary pattern arose in eutherian mammals. Gene duplication is a fundamental process in genome evolution [51, 52]. While some young duplicates are degraded in the process of evolution, some duplicate pairs are able to survive in a long-term. While still controversial, several mechanisms can explain the maintenance of duplicated genes in the genome, including neofunctionalization, subfunctionalization, and increased gene-dosage advantage [51, 53]. A recent theory suggest that young duplicates might be controlled by dosage balance and tight co-regulation of tandem duplicates, allowing the initial survival of the duplicates, followed by its slower adaptation to the genome [54–56]. In our results, we find that repeated gene duplications occurred in a relative quick succession, resulting in several genes closely related. The sequence similarity found in several multigene families are usually a result of conversions and other recombination events, resulting in homogenization of all members of a multigene family, even in the presence of mutations [57]. Yet, the *S100A7* multigene family also displays highly divergent sequences such as that of *M. brandtii_A7(3)*, *M. brandtii_A7(4)* and *M. lucifugus_A7(8)*. Moreover, there is also evidence for the presence of two *S100A7* pseudogenes in *H. sapiens* [20] and several events of gene loss among the eutherian mammals. Therefore, we propose a model of concerted and birth-and-death evolution to explain the obtained results and the evolution of *S100A7* multigene family [57, 58]. This was not the first report of a multigene family with important roles in the immune system having evolved under the birth-and-death model of evolution [59]. In fact, Nei and collaborators suggested that given the importance of some gene families in immune function, natural selection might be a major force of diversification, acting in favor of functional diversification, helping hosts to cope with illness and infection [59].

## Conclusion

In eutherian mammals, multiple duplications, gene loss, recombination and acceleration of point mutations characterize the evolution of the *S100A7* multigene family. Moreover, we suggest a model of concerted and birth-and-death evolution to better explain the evolution of this gene family. Interestingly, several species presented multiple copies of this gene, suggesting that *S100A7* may present a special role in the immune defenses of these species. Nevertheless, future studies are needed to fully elucidate the functional roles of these duplicated *S100A7* genes in the innate immunity of mammals.

## Methods

### Identification of *S100A7* sequences

*S100A7* coding sequences were retrieved from NCBI (http://www.ncbi.nlm.nih.gov) and Ensembl (http://www.ensembl.org/index.html) databases through blastn searches using as reference the *S100A7* and *S100A7A* genes from human. To include all major mammalian orders we analyzed the genomes of several eutherian species: order Artiodactyla, Cetacea, Carnivora, Chiroptera, Perissodactyla, Primates and from the Superorder Afrotheria. According to previous reports, no *S100A7* genes were found in the order Marsupialia, Monotremata and Rodentia [22, 33]. On the other hand, studies also suggest that *S100A15* is not present in human [22, 24]. To confirm the presence of gene loss remnants in both rodents and human, *S100A7* from human and *S100A15* from mouse were used to perform blastn searches in mouse (*GRCm38.p6*) and human (*GRCh38.p12*) genomes, respectively.

Our database search resulted in a final dataset of 82 complete coding sequences, including several duplicated *S100A7* genes in major mammalian orders (Additional file 1). The obtained sequences were aligned with Clustal W [60] implemented in BioEdit v7.2.6.1 using default parameters [61] and manually inspected with the exclusion of gaps and partial sequences (Additional file 2). Nucleotide sequences translation into amino acids was also performed using BioEdit.

### Recombination and phylogenetic analysis

The sequence alignment was screened for recombination using GENECONV, BootScan, MaxChi, Chimaera, SiScan and 3Seq methods implemented in the RDP software (version 4.95) [25] under the following parameters: sequences were set to linear, Bonferroni correction, highest acceptable *P* value of 0.05 and 100 permutations. Only recombination events detected by three or more methods were considered.

To increase the reliability of the phylogenetic analysis, sequences with evidence of recombination were not included in the phylogeny. In order to infer the phylogenetic relationships of the *S100A7* genes in mammals, evolutionary analyses were conducted in MEGA7 [62] using a Maximum Likelihood (ML) tree based on the Tamura-Nei model [63]. The reliability of the clusters was tested by performing the bootstrap test of phylogeny, with 1000 bootstrap replications. *S100A7* coding sequences from *Orycteropus afer afer* (superorder Afrotheria) were used as outgroup.

### Comparative genomics

To infer the duplication history of *S100A7* genes in several mammals, these genes were mapped into their respective chromosomes. For human and mice comparison of *S100* gene loci previous studies were used to identify the location of several *S100* genes [20, 22]. For *Bos taurus*, *Pteropus alecto* and *M. lucifugus* the specific location of *S100A7* genes along with theneighboring genes were collected from NCBI and Ensembl databases using their available genomes (ARS-UCD1.2, ASM32557v1 and Myoluc2.0, respectively). The human *S100A7* gene locus was used as a model of comparison.

### Evolutionary analysis

To test for statistical significance in molecular evolution of *S100A7* bat sequences, a Tajima’s relative test was conducted. For that, the statistical parameters were set as default using MEGA7 [62]. A *P-value* < 0.05 was used to reject the null hypothesis of equal rates between the 3 lineages considered simultaneously (sequence A, sequence B and outgroup).

Selective pressures on specific branches were determined using the phylogenetic tree obtained from the Tamura-Nei model mentioned before and codeml of the PAML 4 package [34]. To test these selective pressures, previously described methodology was used [64, 65]. *P* < 0.05 was used to determine whether or not the alternative hypothesis was significant. We further assessed the strength of natural selection (relaxed or intensified) using DATAMONKEY web server (http://www.datamonkey.org/ [last accessed January 21, 2019] [66, 67]. The *M. lucifugus_A7(8)* and *M. brandtii_A7(4)* branch was the “test” branch, and all the primate branches were assigned as “reference” branches.

## Additional files

Additional file 1: List of the sequences of the *S100A7* genes used in this study. All sequences are available from NCBI and Ensembl databases. (PDF 109 kb)

Additional file 2: Alignment of S100A7 coding sequences from several eutherian mammals. The abbreviations correspond to the ones shown in Additional file 1. Dots = identity with *B. acutorostrata*_A7(1) coding sequence. (PDF 275 kb)

Additional file 3: Alignment between S100A15 and S100A7 proteins. Alignment between two S100A15 proteins from mouse, one S100A15 from *P. troglodytes* and two S100A7 proteins from *H. sapiens* and *P. paniscus*. Dots = identity with *M. musculus*_S100A7_XM_ 006501635.3 protein. (PDF 305 kb)

Additional file 4: Alignment of S100A15 proteins from several eutherian mammals. Dots = identity with *Mus musculus* S10015 protein. (PDF 172 kb)

Additional file 5: Search for the gene loss remnants of *S100A7* and *S100A15* from mouse and human, respectively. (PDF 193 kb)

Additional file 6: Alignment of partial sequences S100A7 from bats. The abbreviations correspond to the following species: *M. lyra* - *Megaderma lyra*; *R. ferrumequinum* - Rhinolopus ferrumequinum; *R. sinicus* - Rhinolophus sinicus; P. patnellii - *Pteronotus parnellii*; H. arimiger - *Hipposideros armiger*; *D. rotundus* - *Desmodus rotundus*. Dots = identity with *M. lucifugus*_A7(1) sequence. (PDF 149 kb)

Additional file 7: Selection (dN/dS) analyses. (PDF 331 kb)

## Abbreviations

MHC: Major histocompatibility complex
ML: Maximum likelihood
Mya: Million years ago
S100A15: S100 Calcium Binding Protein A15
S100A7: S100 Calcium Binding Protein A7
Tas2rs: Taste receptor genes
TLRs: Toll-like receptors
ω: non-synonymous to synonymous substitution ratios
