# Supplementary material for: The evolution of S100A7: an unusual gene expansion in Myotis bats
Source: BMC Evol Biol. 2019 May 14;19:102. doi: 10.1186/s12862-019-1433-0 (PMC6518696; doi:10.1186/s12862-019-1433-0)
Supplement: Supplementary file 1 — List of the sequences of the S100A7 genes used in this study. All sequences are available from NCBI and Ensembl databases. (PDF 109 kb) [file 12862_2019_1433_MOESM1_ESM.pdf]

Additional File 1. List of the sequences of the S100A7 genes, available from NCBI and Ensembl databases and used in this study.

Species name	Gene	Accession no.	Abbreviation	Order
Homo sapiens	S100A7	ENST00000368723	H. sapiens_A7	Primates
	S100A7A	ENST00000368729	H. sapiens_A7A	
Pan troglodytes	S100A7	ENSPTRG00000001350	P. troglodytes_A7	Primates
	S100A7A	ENSPTRG00000001349	P. troglodytes_A7A	
Pan paniscus	S100A7	ENSPPAG00000042606	P. paniscus_A7	Primates
	S100A7A	ENSPPAG00000034917	P. paniscus_A7A	
Gorilla gorilla	S100A7	ENSGGOG00000024101	G. gorilla_A7	Primates
	S100A7A	ENSGGOG00000040685	G. gorilla_A7A	
Nomascus leucogenys	S100A7	ENSNLEG00000010395	N. leucogenys_A7	Primates
	S100A7A	ENSNLEG00000010398	N. leucogenys_A7A	
Papio anubis	S100A7A	ENSPANT00000001109	P. anubis_A7A	Primates
Macaca mulatta	S100A7	ENSMMUG00000047853	M. mulatta_A7	Primates
M. fascicularis	S100A7	ENSMFAG00000033466	M. fascicularis_A7	Primates
	S100A7A	ENSMFAG00000035481	M. fascicularis_A7A	
M. nemestrina	S100A7A	ENSMNEG00000029655	M. nemestina_A7A	Primates
Rhinopithecus bieti	S100A7	ENSRBIG00000036259	R. bieti_A7	Primates
	S100A7A	ENSRBIG00000043734	R. bieti_A7A	
Cebus capucinus	S100A7	ENSCCAG00000028661	C. capucinus_A7(1)	Primates
Saimiri boliviensis	S100A7	ENSSBOG00000018328	S. boliviensis_A7(1)	Primates
Myotis davidii	S100A7 (1)	XM_015559755.1	M. davidii_A7(1)	Chiroptera
	S100A7 (2)	XM_015571787.1	M. davidii_A7(2)	
	S100A7 (3)	XM_015563502.1	M. davidii_A7(3)	
Myotis brandtii	S100A7 (1)	XM_014540742.1	M. brandtii_A7(1)	Chiroptera
	S100A7 (2)	XM_014534771.1	M. brandtii_A7(2)	
	S100A7 (3)	XM_014529525.1	M. brandtii_A7(3)	
	S100A7 (4)	XM_014550114.1	M. brandtii_A7(4)	
	S100A7 (5)	XM_005879336.2	M. brandtii_A7(5)	
Myotis lucifugus	S100A7 (1)	ENSMLUG00000028622	M. lucifugus_A7(1)	Chiroptera
	S100A7 (2)	XM_006095333.3	M. lucifugus_A7(2)	
	S100A7 (3)	ENSMLUG00000027852	M. lucifugus_A7(3)	
	S100A7 (4)	ENSMLUG00000030770	M. lucifugus_A7(4)	
	S100A7 (5)	XM_006095336.3	M. lucifugus_A7(5)	
	S100A7 (6)	ENSMLUG00000028155	M. lucifugus_A7(6)	
	S100A7 (7)	ENSMLUG00000028511	M. lucifugus_A7(7)	
	S100A7 (8)	XM_006097496.3	M. lucifugus_A7(8)	
	S100A7 (9)	XM_006097493.3	M. lucifugus_A7(9)	
	S100A7 (10)	ENSMLUG00000027470	M. lucifugus_A7(10)	
	S100A7 (11)	ENSMLUG00000030259	M. lucifugus_A7(11)	
	S100A7 (12)	ENSMLUG00000023570	M. lucifugus_A7(12)	
	S100A7 (13)	XM_006093750.3	M. lucifugus_A7(13)	
Miniopterus natalensis	S100A7 (1)	XM_016221659.1	M. natalensis_A7(1)	Chiroptera
	S100A7 (2)	XM_016220335.1	M. natalensis_A7(2)	
Eptesicus fuscus	S100A7 (1)	XM_008155898	E. fuscus_A7(1)	Chiroptera
Pteropus vampyrus	S100A7 (1)	ENSPVAG00000006927	P. vampyrus_A7(1)	Chiroptera
Pteropus alecto	S100A7 (1)	XM_006923604	P. alecto_A7(1)	

	S100A7 (2)	XM_006923668	P. alecto_A7(2)	
Rousettus aegyptiacus	S100A7 (1)	XM_016126065.1	R. aegyptiacus_A7(1)	
	S100A7 (2)	XM_016126064.1	R. aegyptiacus_A7(2)	Chiroptera
	S100A7 (3)	XM_016140198.1	R. aegyptiacus_A7(3)	
Equus caballus	S100A7	XM_014739696	E. caballus_A7(1)	Perissodactyla
Equus asinus	S100A7	XM_014864979	E. asinus_A7(1)	Perissodactyla
Bos taurus	S100A7 (1)	NM_174596.2	B. tauros_A7(1)	
	S100A7 (2)	XM_002686002.5	B. tauros_A7(2)	Artiodactyla
	S100A7 (3)	XM_024989827.1	B. tauros_A7(3)	
B. indicus	S100A7 (1)	XM_019955344.1	B. indicus_A7(1)	
	S100A7 (2)	XM_019958349.1	B. indicus_A7(2)	Artiodactyla
	S100A7 (3)	XM_019958159.1	B. indicus_A7(3)	
Bison bison	S100A7 (1)	XM_010857440.1	B. bison_A7(1)	Artiodactyla
	S100A7 (2)	XM_010855847.1	B. bison_A7(2)	
Bubalus bubalis	S100A7 (1)	XM_006051696.2	B. bubalis_A7(1)	
	S100A7 (2)	XM_025287355.1	B. bubalis_A7(2)	Artiodactyla
	S100A7 (3)	XM_025287356.1	B. bubalis_A7(3)	
Pantholops hodgsonii	S100A7 (1)	XM_005974980.1	P. hodgsonii_A7(1)	Artiodactyla
	S100A7 (2)	XM_005974979.1	P. hodgsonii_A7(2)	
Capra hircus	S100A7 (1)	KC345019.1	C. hircus_A7(1)	
	S100A7 (2)	XM_018046586.1	C. hircus_A7(2)	Artiodactyla
	S100A7 (3)	XM_005677509.2	C. hircus_A7(3)	
Ovies aries	S100A7 (1)	XM_012132139.2	O. aries_A7(1)	Artiodactyla
	S100A7 (2)	XM_004002524.3	O. aries_A7(2)	
Sus scrofa	S100A7 (1)	XM_021090794.1	S. scrofa_A7(1)	Artiodactyla
	S100A7 (2)	XM_021090793.1	S. scrofa_A7(2)	
Balaenoptera acutorostrata	S100A7 (1)	XM_007178590.1	B. acutorostrata_A7(1)	Cetacea
Camelus dromedarius	S100A7 (1)	XM_010980642.1	C. dromedarius_A7(1)	Artiodactyla
Camelus ferus	S100A7 (1)	XM_006177172.2	C. ferus_A7(1)	Artiodactyla
Camelus bactrianus	S100A7 (1)	XM_010954054.1	C. bactrianus_A7(1)	Artiodactyla
Vicugna pacos	S100A7 (1)	XM_006214733.2	V. pacos_A7(1)	Artiodactyla
Ailuropoda melanoleuca	S100A7 (1)	XM_002922540.3	A. melanoleuca_A7(1)	Carnivora
Ursus arctos	S100A7 (1)	XM_026485405.1	U. arctos_A7(1)	Carnivora
Ursus maritimus	S100A7 (1)	XM_008696673.1	U. maritimus_A7(1)	Carnivora
Callorhinus ursinus	S100A7 (1)	XM_025858783.1	C. ursinus_A7(1)	Carnivora
Orycteropus afer afer	S100A7 (1)	XM_007948588.1	O. afer_A7(1)	Afrotheria
	S100A7 (2)	LOC103203527	O. afer_A7(2)	
Loxodonta africana	S100A7 (1)	XM_007948587.1	L. africana_A7(1)	Afrotheria
