# Supplementary material for: The evolution of S100A7: an unusual gene expansion in Myotis bats
Source: BMC Evol Biol. 2019 May 14;19:102. doi: 10.1186/s12862-019-1433-0 (PMC6518696; doi:10.1186/s12862-019-1433-0)
Supplement: Supplementary file 2 — Alignment of S100A7 coding sequences from several eutherian mammals. The abbreviations correspond to the ones shown in Additional file 1. Dots = identity with B. acutorostrata_A7(1) coding sequence. (PDF 275 kb) [file 12862_2019_1433_MOESM2_ESM.pdf]

**Additional File 2. Alignment of *S100A7* coding sequences from several eutherian mammals.** The abbreviations correspond to the ones shown in Additional File 1. Dots = identity with B. acutorostrata_A7(1) coding sequence.

```
                                  10        20        30        40        50        60        70        80        90       100
                         ....|....|....|....|....|....|....|....|....|....|....|....|....|....|....|....|....|....|....|....|
B. acutorostrata_A7(1)   ATGAGCAGC---------ACTGAGGCTGAGAAGACCACGATAGGCACGAGTGACCTGTCTCACAAATACTCGGGGCCCGAAGACACCATCGAGAAGGGGG
B. tauros_A7(1)          .........---------T..C..CT....C..G...TT.C..A.TT..TCA......T...............AT....T..................A..
B. tauros_A7(2)          ......G..---------TT.C..CT....C..G...TT.C..A.TT..TCA......T...............AT....T..................A..
B. tauros_A7(3)          ......G..---------TT.C.CCT....C..G...TT.C..A.TT..TCA......T................T....T..................A..
B. indicus_A7(1)         ......G..---------T..C..CT....C..G...TT.C..A.TT..TCA......T...............AT....T..................A..
B. indicus_A7(2)         ......G..---------TT.C..CT....C..G...TT.C..A.TT..TCA......T...............AT....T..................A..
B. indicus_A7(3)         ......G..---------TT.C.CCT....C..G...TT.C..A.TT..TCA......T...............AT....T..................A..
B. bison_A7(1)           ......G..---------TT.C..CT....C..G...TT.C..A.TT..TCA......T...............AT....T..................A..
B. bison_A7(1)           .........---------T..C..CT....C..G..GTT.C..A.TT..TCA......T...............AT....T..................A..
B. bubalis_A7(1)         .......T.---------T..C..CT....C..G....T.CC.A.TT..TCA.A....T...............AT....T..................A..
B. bubalis_A7(2)         ......GT.---------T..C..CT....C..G...TTTC..ATTT..TCA.T....T...............A.A...T..................A..
B. bubalis_A7(3)         ......GT.---------T..C..CT....C..G...TT.C..A.TT..TCA.T....T...............A.A...T..................A..
P. hodgsonii_A7(1)       ......G..---------T..C..CTG...C..G...TTTC..C.TT..TCA......T.......C....A..A.....T..T...............A..
P. hodgsonii_A7(2)       ......G..---------T..C..CTG...C..G...TTTC..C.TT..TCA......T.......C....A..A.....T..T...............A..
C. hircus_A7(1)          .........---------T..C..CTG...C..G...TTTC..C.TT..TC.......T.......C.......A.....T..................A..
C. hircus_A7(2)          ......G..---------T..C..CTG...C..G...TTTC..C.TT..TC.......T.......C.......A.....T..................A..
C. hircus_A7(3)          ......G..---------T..A..CTG...C..G...TTTC..C.TT..TC.......T.......C.......A.....T..................A..
O. aries_A7(1)           ......G..---------T..C..CTG...C..G...TTTC..C.TT..TC.......T.......C.......A.....T..................A..
O. aries_A7(2)           ......G..---------T..C..CTG...C..G...TTTC..C.TT..TC.......T.......C.......A.....T..................A..
C. dromedarius_A7(1)     ...G.A...---------....C...C......T..GT...G....T..TC...A...T......G...A.C...AAA..C...........C...CC..
C. ferus_A7(1)           ...G.A...---------....C...C......T..GT...G....T..TC...A...T......G...A.C...AAA..C...........C...CC..
C. bactrianus_A7(1)      ...G.A...---------....C...C......T..GT...G....T..TC...A...T......G...A.C...AAA..C...........C...CC..
V. pacos_A7(1)           ...G.A...---------....C...C......T..GT...G....T..TC...A...T......G...A.C...AG...C...........C...CC..
S. scrofa_A7(1)          .......C.---------...CC...C......T...T...G.A.....TC.......TC.......T.A.A.ACGA.AGT........G..C....A..
S. scrofa_A7(2)          .......C.---------...CC...C......T...T...G.A.....TC.......TC.......T.A.A.ACGA.AGT........G..C....A..
A. melanoleuca_A7(1)     .........---------..ACGA.........TT.GT.G.G....TC.T...G....T......TG..A.CA...A.C.T........G..C...CCA.
U. arctos_A7(1)          .........---------..ACGA.........TT.GT.G.G....TCCTC..G....T......TG..A.CA...A.C.T.......CG..C...CC..
U. maritimus_A7(1)       .........---------..ACGA.........TT..T.G.G....TCCTC..G....T......TG..A.CA...A.C.T.......CG..C...CC..
C. ursinus_A7(1)         .........---------..AC.A.........TT.GT.G.G....TC.TC..A..A.T......C...A.CA...A.C.T........G..C...TCA.
E. asinus_A7(1)          .......AA---------.....A........GC.T.TGT...T....TA.TC..G....T..........A.C..A.GT..T....TG..T..C...CCA.
E. caballus_A7(1)        ......GAA---------.....A........GC.T.TGT...T....TA.TC..G....T..........A.C..A.GT..T....TG..T..C...CCA.
M. lucifugus_A7(1)       .....TCAA---------...C.A.T.......T...T...G.A.TT.GT..T.....TC.........A.CAAA.....T....AG..T..C...C...
M. lucifugus_A7(2)       ......CAT---------...CCA.T...AC..T..GT...G.A.TT..TC.T.....TC.........A.CAAA..T..T....AG..T..C...C...
M. lucifugus_A7(3)       ......CAT---------...CCA.T...AC..T..GT...G.A.TT..TCAT.....TC.........A.CAAA..T..T....AG..T..C...C...
M. lucifugus_A7(4)       ......CAT---------...CCA.T...AC..T..GT...G.A.TT..TC.T.....TC.........A.CAAA..T..T....AG..T..C...C...
M. lucifugus_A7(5)       ......CAT---------...CCA.T...AC..T..GT...G.A.TT..TC.T.....TC.........A.CAAA..T..T....AG..T..C...C...
M. lucifugus_A7(6)       ......CAT---------...CCA.T...AC..T...T...G.A.TT..TC.T.....T..........A.CAAA..T..T....AG..T..C...CA..
M. lucifugus_A7(7)       ......CAT---------...CCA.T...AC..T..GT...G.A.TT..TC.T.....TC.........A.CAAA..T..T....AG..T..C...C...
M. lucifugus_A7(8)       ...G..CAT---------...C.A.....AGT.G.T.T...G.A.TT.GT.....A..T.....G....A.CAAA.....T....AG..TA.C...AA..
M. lucifugus_A7(9)       ......CAT---------...CCA.T...AC..T..GT...G.A.TT..TC.T.....TC.........A.CAAA..T..T....AG..T..C...CA..
M. lucifugus_A7(10)      ......CAT---------...CCA.T...AC..T..GT...G.A.TT..TC.T.....TC.........A.CAAA..T..T....AG..T..C...C...
M. lucifugus_A7(11)      ......CAT---------...CCA.T...AC..T..GT...G.A.TT..TC.T.....TC.........A.CAAA..T..T....AG..T..C...C...
M. lucifugus_A7(12)      ......CAT---------...CCA.T...AC..T..GT...G.A.TT..TC.T.....TC.........A.CAAA..T..T....AG..T..C...CA..
M. lucifugus_A7(13)      ......CA.---------...C.C.......G.TA.GT...GAC..T..TC..T....TC.........AGCAAA..T..T....TG..T.CC...CC..
M. brandtii_A7(4)        ...G..CAT---------....TA.....AC..G...T...G.A.TT..TC....A..T.....G....A.CAAA..T..T....AG..TA.C...AA..
M. brandtii_A7(1)        .....TCAA---------...C.A.T.......T...T...G.A.TT.GTC.T.....TC.........A.CAAA.....T....AG..T..C...C...
M. brandtii_A7(5)        ......CA.---------...C.C.........TATGT...GAC..T..TC..T....TC.........AGCAAA..T..T....TG..T.CC...CC..
M. brandtii_A7(2)        ......CAT---------...CCA.T...AC..T.TGT...G.A.TT..TC.T.....TC.........A.CAAA..T..T....AG..T..C...CA..
M. davidii_A7(2)         ......CAA---------...C.A.T.......T..GT...G.A.TT.GTC.T.....TC.........A.CAAA..T..T....AG..T..C...C...
M. davidii_A7(1)         ......CA.---------...A.C.........TA.GT...GAC..T..TCA.T....TC.........AGCAAA..T..T....TG..T.CC...CC..
M. davidii_A7(3)         ......CA.---------...CCA.T...AC..T..GT.GAG.A.TT..TC.T.....TC.........A.CAAA..T..T....AG..T..C...CCC.
M. natalensis_A7(1)      ......CA.---------...CCA.........T..TT...G.A..T.GTC.T.....TC.........A.CAAA..T..T....AG..T..C...CCA.
M. natalensis_A7(2)      ......CC.---------...CCA.........T..TT...G.A..T.GTC.T.....TC.........A.CAAA.....T....AG..T..C...CCA.
E. fuscus_A7(1)          ......TA.---------...CCA.........TA.GT...GA...T.GTC.......TC......C..AGCAAA..G..C....TG..T..CG..CC..
R. aegyptiacus_A7(1)     ......TA.---------..GC..............GT...G....T..T........TC....C....GTCAAA.....C....TG..G..C...CCC.
R. aegyptiacus_A7(3)     ......TA.---------..AC..............GT...G....T..T........TC....C....GTCAAA.....C....TG..G..C...CCC.
R. aegyptiacus_A7(2)     ......CA.---------..GC........G..G..GT...G....T..TC.......TC....CG...GTCAAA.....C....TG..G..C...CCC.
P. vampyrus_A7(1)        ......CA.---------..GC...........G..GT...G.A..T..TCA.G....TC....CG...A.CAAA.....C....TG.....C...TC..
P. alecto_A7(1)          ......CA.---------..GC...........G..GT...G.A..T..TC..G....TC....CG...A.CAAA.....C....TG.....CG..CC..
P. alecto_A7(2)          ......CA.---------..GC...........G..GT...G.A..T..TC..G....TC....CG...A.CAAA.....C....TG.....CG..CC..
H. sapiens_A7            .......A.---------...C.A.......G.T...TA.......T..TC...A...T..........A.CA.A.GT..T....AG..T......CCAA
H. sapiens_A7A           .......A.---------...C.A.......G.T...TA.......T..TC...A...T..........A.C..A.GT..T.G..AG..T......CCAA
P. troglodytes_A7        .......A.---------...C.A.......G.T...TA.......T..TC...A...T..........A.CA.A.GT..T....AG..T..C...CCAA
P. troglodytes_A7A       .......A.---------...C.A.......G.T...TA.......T..TC...A...T..........A.CA.A.GT..T....AG..T..C...CCAA
P. paniscus_A7           .......A.---------...C.A.......G.T...TA.......T..TC...A...T..........A.CA.A.GT..T....AG..T..C...CCAA
P. paniscus_A7A          .......A.---------...C.A.........T...TA.......T..TC...A...T..........A.CA.A.GT..T....AG..T..C...CCAA
G. gorilla_A7            .......A.---------...C.A.......G.T...TA.......T..TC...A...T..........A.CA.A.GT..T....AG..T..C...CCAA
G. gorilla_A7A           .......A.---------...C.A.......G.T...TA.......T..TC...A...T..........A.CA.A.GT..T....AG..T..C...CCAA
N. leucogenys_A7         ....A..A.---------...C.A.......C.T..GTA.......T..TC...A...T..........A.CA.A.GTA.T....AG..T..C...CCAA
N. leucogenys_A7A        ....A..A.---------...C.A.......C.T...TA.......T..TC...A...T..........A.CA.A.GT..T....AG..T..C...CCAA
P. anubis_A7A            .......A.---------...C.A.......C.T...TA.......T..TC...A...T..........A.CA.A.GT..T....AG..T..C...CCAA
M. mulatta_A7            .......A.---------...C.A.......C.T...TA.......T..TC...A...T..........A.CA.A.GT..T....AG..T..C...CCAA
M. fascicularis_A7A      .......A.---------...C.A.......C.T...TA.......T..TC...A...T..........A.CA.A.GT..T....AG..T..C...CCAA
M. fascicularis_A7       .......A.---------...C.A.......C.T...TA.......T..TC...A...T..........A.CA.A.GT..T....AG..T..C...CCAA
M. nemestina_A7A         .......A.---------...C.A.......C.T...TA.......T..TC...A...T..........A.CA.A.GT..T....AG..T..C...CCAA
R. bieti_A7A             .......A.---------...C.A.......C.T...TA.......T..TC...A...T..........A.CA.A.GT..T....AG..T..C...CCAA
R. bieti_A7              .......A.---------...C.A.......C.T...TA.......T..TC...A...T..........A.CA.A.GT..T....AG..T..C...CCAA
C. capucinus_A7(1)       .........---------...C.A.........T...TAG.G....T..TCA..A...T..........A.C..A.GT..C....AG..T..C...CCCA
S. boliviensis_A7(1)     .........---------...C.A.........T...TAG.G....T..TC...A...T..........A.C..AAAT..C....AG..T..C...CCCA
O. afer_A7(1)            .......T.---------...C.A.............T...T.A..T..TCTTA..T.T.T.T.....TA.T..AAT...T....AG..T..C...CCT.
O. afer_A7(2)            .......A.---------.G.A.A......C...T..T...C.A..T..TCA....C.T...T..G..TA.T..AAT...T....AG.....CG..CCT.
L. africana_A7(1)        ---------CTTGGTGATTTCCTA.G...A..TTTGGTCC.GA..TT.GT...A..A.T...TC.....A.C...TAT..T..T.AG...A.C.G..AAA

                                 110       120       130       140       150       160       170       180       190       200
```

```
                          ....|....|....|....|....|....|....|....|....|....|....|....|....|....|....|....|....|....|....|....|
B. acutorostrata_A7(1)    ACCCGCCGAAGCTGTTGAAGGAGAATTTCCCCAACTTGCTCAGTGCCCGTACCAAAATGGGCACAGATTACTTGTCCACTGTCTTTGAGAAAAAGGACAA
B. tauros_A7(1)           ...T..T.CG....A.......C..C...........C...G.....T..GAG....G.....G..............A.A...........C.A.....
B. tauros_A7(2)           ...T..T.CG....A..........C...........C.........T..GAG....G.....G.C.A.........GA.A...................
B. tauros_A7(3)           ...T..T.CG....A..........C...........C.........T..GAG....G.....G.C.A.........GA.A...................
B. indicus_A7(1)          ...T..T.CG....A..........C...........C...G.....T..GAG....G.....G..............A.A...........C.A.....
B. indicus_A7(2)          ...T..T.CG....A..........C...........C.........T..GAG....G.....G.C.A.........GA.A...................
B. indicus_A7(3)          ...T..T.CG....A..........C...........C...G.....T..GAG....G.....G.C.A.........GA.A...................
B. bison_A7(1)            ...T..T.CG....A..........C...........C.........T..GAG....G.....G.C.A.........GA.A...................
B. bison_A7(1)            ...T..T.C.....A.......T..C...........C.........T..GAG....G.....G..............A.A...........C.A.....
B. bubalis_A7(1)          ...T..T.CG....A..........C...........C...G.....T..GAG....G.....G.C.A........TGA.A...................
B. bubalis_A7(2)          ...T..T.CG....A..........C...........C...G.....T..GAG....G.....G..............A.A...........C.A.....
B. bubalis_A7(3)          ...T..T.CG....A..........C....AG.....C.........T..GAG....G.....G.C.A.........GA.A...................
P. hodgsonii_A7(1)        C..T..T.C.....C..........C...........C.........T..GAG....G.....G..............A.A...................
P. hodgsonii_A7(2)        C..T..T.C.....C..........C...........C.........T..GAG....G.....G.C............A.A...................
C. hircus_A7(1)           C..T..T.C.....C..........C...........C.........T..GAG....G.....G..............A.A...................
C. hircus_A7(2)           C..T..T.C.....C..........C...........C.........T..GAG....A....CG.C............A.A...................
C. hircus_A7(3)           C..T..T.C.....C..........C...........C.........T..GAG....G.....G..............A.A...................
O. aries_A7(1)            C..T..T.C.....C.......T..C...........C.........T..GAG....G....CG.C............A.A...................
O. aries_A7(2)            C..T..T.C.....C.......T..C...........C.........T..GAG....G.....G..............A.A.T.................
C. dromedarius_A7(1)      G.GT..A....A..C..........C...........C.........T..GAA....G.....A..............ACA........C..........
C. ferus_A7(1)            G.GT..A....A..C..........C...........C.........T..GAA....G.....A..............ACA........C..........
C. bactrianus_A7(1)       G.GT..A....A..C..........C...........C.........T..GAA....G.....A..............ACA........C..........
V. pacos_A7(1)            G.GT..A....A..C..........C...........C.........T..GAT....G.....A..............ACA.......AC..........
S. scrofa_A7(1)           G..T..T.......C..C...................C.........T..GA.....AT..TGTC......C..G.T.A.A........C..........
S. scrofa_A7(2)           G..T..T.......C..C...................C.........T..GA.....AT..TGTC......C..G.T.A.A........C..........
A. melanoleuca_A7(1)      C.TT..T....A..C.CC................G..C.........T..GA.....A........C.....G.G...A.A...............G...
U. arctos_A7(1)           CATT..T....AC.C.CC.....G..........G..C.....C...T..GA.....A........C.....G.G...A.A...............G...
U. maritimus_A7(1)        CATT..T....AC.C.CC.....G..........G..C.....C...T..GA.....A........C.....G.G...A.A...............G...
C. ursinus_A7(1)          C.TT..T....A..C.CC................G..C....A....T..GA.....A.C............G.G...A.AC...........G..G...
E. asinus_A7(1)           G..T.TT....A..C..C....C..............C..TGC....T..GA.....A................G...A...........G........
E. caballus_A7(1)         G..T.TT....A..C..C....C..............C..TGC....T..GA.....A................G...A...........G........
M. lucifugus_A7(1)        G..T..T....A..C...G............T.TA..C....AA...T..GA.....A.....AT....T....GA.CA.A........G..........
M. lucifugus_A7(2)        G..T..T....A..C...G............T.....C....TG..TT..GAA....A.....A.....T....G..CA.A........G.TG.......
M. lucifugus_A7(3)        G..T..T....A..C...G............T.....C....AC..TT..GAA....A.....A.....T....G..CA.A........G.CG.......
M. lucifugus_A7(4)        G..T..T....A..C...G............T.....C....AG..TT..GAA....A.....AT....T....G..CA.A.......AG.CC.......
M. lucifugus_A7(5)        G..T..T....A..C...G............T.....C....TG..TT..GAA....A.....AT....T....G..CA.A........G.CG.......
M. lucifugus_A7(6)        G..T..T....A..C...G............T.....C....AG..TT..GAA....AA....A.....T....G..CA.A........G.CG.......
M. lucifugus_A7(7)        G..T..T....A..C...G............T.....C....TG..TT..GAA....A.....A.....T....G..CA.A........G.TG.......
M. lucifugus_A7(8)        G..T..T..G.A..C...G........A...TC....CA...A...TT..GAA....G....GT.....T....GGGGA.A..........C........
M. lucifugus_A7(9)        G..T..T....A..C................G.....C....AG..TT..GAA....A.....A.....T....G..CA.A........G.CG.......
M. lucifugus_A7(10)       G..T..T....A..C...G............T.....C....AC..TT..GAA....A.....A.....T....G..CA.A........G.CG.......
M. lucifugus_A7(11)       G..T..T....A..C...G............T.....C....TG..TT..GAA....A.....A.....T....G..CA.A........G.CG.......
M. lucifugus_A7(12)       G..T..T....A..C................T.....C....AG..TT..GAA....A.....A.....T....G..CA.A........G.CG.......
M. lucifugus_A7(13)       G..T..T..T.T..C................T.....CG...AG.A.T..GA...G.CG.....AC............CA..........G.......TC.
M. brandtii_A7(4)         G..TT.T..G.A..C...G........AT..TC....C....AC..TT..GAA..T.G.....T.....T....GG.GA.A..........C........
M. brandtii_A7(1)         G..T..T....A..C...G............T.TA..C....AA...T..GA.....A.....AT....T....GA.CA.A........G..........
M. brandtii_A7(5)         G..T..T..T.T..C................T.....CG...AG.G.T..GA...G.CG.....ACA...........CAA.........G.......TCT
M. brandtii_A7(2)         G..T..T....A..C...G............T.....C....AG..TT..GAAG...AA...GA.C...T....G..CA.A........G.CG.......
M. davidii_A7(2)          G..T..T....A..C................T.TA..C....AG..TT..GA.....A.....AT....T....GA.CA.A........G..........
M. davidii_A7(1)          G..T..T..T.T..C................T.....CG...AG.G.T..GA...GCG.....ACA...........CA..........G.......TCG
M. davidii_A7(3)          G..T..T....A..C................T.....C....AG..TT..GAA....A.....A.....T....G..CA.A........G.TG.......
M. natalensis_A7(1)       G..T..T....A..C......................C....AG..TT..GAAG.G.A.....A.A...........T..A........G.CTC......
M. natalensis_A7(2)       G..T..T....A..C......................C....AG...T..GAAGT..A.....A.A...........T..A........G.CTC......
E. fuscus_A7(1)           G..T..T..T.T..C................T.....TG...AG...T..GAG.G..G.....A.A...........CA..........G.......TC.
R. aegyptiacus_A7(1)      G..T.ATC...A..C..C....C..........C...C.........T..GA.....A.....A.............CACA....C...G.T......C.
R. aegyptiacus_A7(3)      G..T.ATC...A..C..C....C..........C...C.........T..GA.....A.....A.............CACA....C...G.T......C.
R. aegyptiacus_A7(2)      G..T.ATC...A..C..C....C..........C...C.........T..GA.....A.....A..........G..CACA....C...G.T......C.
P. vampyrus_A7(1)         G..T.ATC...A..C..C...............C...C.....C...T..GA.....A.....A.............CACA....C...G.T......C.
P. alecto_A7(1)           G..T.A.C...A..C..C...............C...C.....C...T..GG.....A.....A.............CACA....C...G.T......C.
P. alecto_A7(2)           G..T.A.C...A..C..C...............C...C.....C...T..GA.....A.....A.............CACA....C...G.T......C.
H. sapiens_A7             G..T..T..C.A..A..........C...........C..T......T..GA.....A.......A.....C.CG..GA.....................
H. sapiens_A7A            G..T..T..C.A..A..........C........T..C.........T..GA.....A.....T.C.....C.CG.........................
P. troglodytes_A7         G..T..T..CCA..A..........C...........C..T......T..GA.....A.......A.....C.CG...A..................T..
P. troglodytes_A7A        G..T..T..C.A..A..........C........T..C.........T..GA.....A.....T.C.....C..G.........................
P. paniscus_A7            G..T..T..CCA..A..........C...........C..T......T..GA.....A.......A.....C.CG...A..................T..
P. paniscus_A7A           G..T..T..C.A..A..........C........T..C.........T..GA.....A.....T.C.....C..G.........................
G. gorilla_A7             G..T..T..C.A..A..........C...........C..T......T..GA.....A.......A.....C.CG...A.....................
G. gorilla_A7A            G..T..T..C.A..A..........C........T..C.........T..GA.....A.....T.C.....C..G.........................
N. leucogenys_A7          G..T..T..C.A..A..........C...........C..T......T..GA.....A.......A.....C..G...A...............A.....
N. leucogenys_A7A         G..T..T..C.A..A..........C...........C..T......T..GA.....A.......A.....C..G...A...............A.....
P. anubis_A7A             G..T..T..C.A..A..........C...........C.........T..GA.....A.....T.C.....C..G...G...........G.........
M. mulatta_A7             G..T..T..C.A..A..........C...........C.........T..GA.....A.....T.C.....C..A...A...........G.........
M. fascicularis_A7A       G..T..T..C.A..A..........C...........C.........T..GA.....A.....T.C.....C..A...A...........G.........
M. fascicularis_A7        G..T..T..CAA..A....A.....C...........C.........T..GA.....A.....T.C.....C..A...A...........G.........
M. nemestina_A7A          G..T..T..C.A..A..........C...........C.........T..GA.....A.....T.C.....C..A...A...........G.........
R. bieti_A7A              G..T..T..C.A..A..........C...........C.........T..GA.....A.....T.C.....C..A...A...........G.........
R. bieti_A7               G..T..T..C.A..A..........C...........C.........T..GA.....A.....T.C.....C..A...A...........G.........
C. capucinus_A7(1)        G..T..T..C.A..A..........C...........C.........T..GA.....A.......A.....C..G...A..C..................
S. boliviensis_A7(1)      G..T..T..C.A..A..........C...........C.........T..GA.....A.......A.....C..G...A..C..................
O. afer_A7(1)             G..T..T....A..A......................C.........T..GAG....G.......A........G...ACA...................
O. afer_A7(2)             G..T..T....A..A......................C.........T...AG....G................G...ACA...................
L. africana_A7(1)         ...T..T......AC..........C...........C....A..A.T..GAA.G..G.....A.........AG...A...............A.....

                                   210       220       230       240       250       260       270       280       290       300
                          ....|....|....|....|....|....|....|....|....|....|....|....|....|....|....|....|....|....|....|....|
B. acutorostrata_A7(1)    GAATAAGGACAAGAAGGTTGACTTTTCCGAGCTCCTCTCCTTGCTGGGGGATATAGCCACAGACTACCACAATCAGGGCCACAGAGCGCCACCCTGTTCT
B. tauros_A7(1)           ..........CG....A..........T...T....G..........C...C..............T.....C..CA.....G....A.AG.T.......
B. tauros_A7(2)           ................A..........T...T....G..........C...C...........T........C..CA.....G....A.AG.T.......
B. tauros_A7(3)           ................A..........T...T....G..........C...C...........T........C..CA.....G....A.AG.T.......
B. indicus_A7(1)          ......R...CG....A..........T...T....G..........C...C..............T.....C..CA.....G....A.AG.T.......
B. indicus_A7(2)          ................A..........T...T....G..........C...C...........T........C..CA.....G....A.AG.T.......
B. indicus_A7(3)          ................A..........T...T....G..........C...C...........T........C..CA.....G....A.AG.T.......
B. bison_A7(1)            ................A..........T...T....G..........C...C.....................C..CA.....G....A.AG.T.......
B. bison_A7(1)            ..........CG....A..........T...T....G..........C...C...A.................C..CA....TG....A.AG.T.......
B. bubalis_A7(1)          ..........C...G.A..........T...T....G..........T...C.....................C..CA.....G....AGAG.T.......
B. bubalis_A7(2)          ..........CG....A..........T...T....G..........C...C...A..........T.....C..CA.....G...ACAAG.T.......
```

```
B. bubalis_A7(3)          ..........C...G.A..........T...T....G..........C...C....................C..CA.....G....AGAG.T.......
P. hodgsonii_A7(1)        ..........C.....A..........T...T....G..........C...C......T.......T.....C..CA.....G...A.GAG.T.......
P. hodgsonii_A7(2)        ..........C.....A..........T...T....G..........C...C......T.......T.....C..CA.....G...A.GAG.T.......
C. hircus_A7(1)           ..........C.....A..........T...T....G..........C...C......T.......T.....C..CA....TG...A.GAG.T.......
C. hircus_A7(2)           ..........C.....A..........T...T....G..........C...C......T.......T.....C..CA.....G.....GAG.T.......
C. hircus_A7(3)           ..........C.....A..........T...T....G..........C...C......T.......T.....C..CA....TG...A.GAG.T.......
O. aries_A7(1)            ..........C.....A..........T...T....G..........CA..C......T.......T.....C..CA.....G.....GAG.T.......
O. aries_A7(2)            ..........C.....A..........T...T....G..........C...C......T.......T.....C..TA....TG...A.GAG.T.......
C. dromedarius_A7(1)      ....GG..........A..............T.T.............CC.........GTC...........A...A....AG...GAGAG.T.......
C. ferus_A7(1)            ....GG..........A..............T.T.............CC.........GTC...........A...A....AG...GAGAG.T.......
C. bactrianus_A7(1)       ....GG..........A..............T.T.............CC.........GTC...........A...A....AG...GAGAG.T.......
V. pacos_A7(1)            ....GG..........A..............T.T.............CC.........GTC...........A...A.....G...G.GAG.T......C
S. scrofa_A7(1)           ..........C...G.A....G.....T...T.T..G..............C........G.G.........A..CA....TCA.CAAGA..T....C..
S. scrofa_A7(2)           ..........C...G.A....G.....T...T.T..G..............C..........GT........A..CA....TCA..AAGA..T....C..
A. melanoleuca_A7(1)      ....GG...T......A....G......C..T.TTCA..............A...A..GTG....T......A...A.....G....AG.CTG...G...
U. arctos_A7(1)           ....GG...T......A....G......A..T.TT.A..............A...A...TG....T......A...A.....G....AG.CT........
U. maritimus_A7(1)        ....GG...T......A....G......A..T.TT.A..............A...A...TG....T......A...A.....G....AG.CT........
C. ursinus_A7(1)          ....GG...T......A....G......A..T.TT.A..T.GCTG....A.A..TAG.CAC....T......A...A.....G....AG.CT...C....
E. asinus_A7(1)           .....GA.........A..............T.T.....T...........C....................A...A....TG....A...G........
E. caballus_A7(1)         .....GA.........A..............T.T.....T...........C....................A...A....TG....A...G........
M. lucifugus_A7(1)        .........T......A.C..G.........T.T..G..GG..T....A.TC........G...............A.....G..A.C..G.T......C
M. lucifugus_A7(2)        ....T.C..T......A.C..G.........T.T..G..GG..T....A.TC........G..............CA.....G....C..G.T......A
M. lucifugus_A7(3)        ....C.C..T......A.C............T.T..G..GG..T....A.TC........G..............CA.....G....C..G.T.......
M. lucifugus_A7(4)        ......C.........A.C..G.........T.T..G..GG..T....A.TC........G..............CA.....G....C..G.T.......
M. lucifugus_A7(5)        ....C.C.........A.C..G.........T.T..G..GG..T....A.TC........G..............CA.....G....C..G.T.......
M. lucifugus_A7(6)        ....C.C..T......A.C..G.........T.T..G..GG..T....A.TC........G..............CA.....G....C..G.T.......
M. lucifugus_A7(7)        ....C.C..T......A.C..G.........T.T..G..GG..T....A.TC........G..............CA.....G....C..G.T.......
M. lucifugus_A7(8)        ...C.T...T......A.CTCG...ATT...T.T..A..GG..T....A.TCC....TGTG.........G..A.TA.....G....C....T.......
M. lucifugus_A7(9)        ....C.C..T......A.C..G.........T.T..G..GG..T....A.TC........G..............CA.....G....C..G.T.......
M. lucifugus_A7(10)       ....C.C.........A.C..G.........T.T..G..GG..T....A.TC........G..............CA.....G....C..G.T.......
M. lucifugus_A7(11)       ....C.C..T......A.C............T.T..G..GG..T....A.TC........G...........C..CA.....G....C..G.T.......
M. lucifugus_A7(12)       ....C.C.........A.C..G.........T.T..G..GG..T....A.TC........G..............CA.....G....C..G.T.......
M. lucifugus_A7(13)       ...CG....T......A.CC.G.....G...T.T..G.G............C.......TG..........GG...A.....G....C..G.........
M. brandtii_A7(4)         ...C.T...T......A.CTC....ATT...T.T..G..GG..T....A.TCC....TGTG........TG..A.TA.....G....C....T.......
M. brandtii_A7(1)         .........T......A.C..G.........T.T..G..GG..T....A.TC........C...............A.....G....C..G.T......C
M. brandtii_A7(5)         ...CG....T......A.CC.G.....G...T.T..G.G....T.......C.......TG..........GG...A....TG....CG.G.........
M. brandtii_A7(2)         ....C.C..T......A....G.........T.T..G..GG..T....A.TC........G..............TA.....G....C....T.......
M. davidii_A7(2)          .........T......A.C..G.........T.T..G..GG..T....A.TC........C...............A.....G....C..G.........
M. davidii_A7(1)          ...CG....T....T.A.CC.G.....G...T.T..G.G....T.A.....C.......TG........T.GG...A.....G....C..G.........
M. davidii_A7(3)          .........T......A.C............T.T..G.TGG..T....A.TC........G..............CA.....G.C..C..G.T.......
M. natalensis_A7(1)       ....GCA..T......A.CC.G..C..T...T.T..G...G..A....C.GC..T....AC.........G.G...A.....G.............C..C
M. natalensis_A7(2)       ....GCA..T......A.CC.G..C......T.T..G...G..A....C.GC..T....AC.........G.G...A.....G.............C...
E. fuscus_A7(1)           .........T......A.CC.G.....G...T.T..G.G....G.......C.......TG..........GG...A.....G....C.TG........G
R. aegyptiacus_A7(1)      ...C.....T......A.C..G..C......T....G...G.CG.......C..C.................A...A.....G.......GG....C..C
R. aegyptiacus_A7(3)      ...C.....T......A.C..G..C......T....G...G.CG.......C..C.................A...A.....G.......GG....C..C
R. aegyptiacus_A7(2)      ...C.....T......A.C..G..C......T....G...G.CG.......C..C.....G...........A...A.....G.......GG....C..C
P. vampyrus_A7(1)         ...C..A..T......A.C..G..C......T....G...G.CG.......C..C.................A...A.....G....A..GG....C..C
P. alecto_A7(1)           ...C..A..T......A.C..G..C......T....G...G.CA.A.....C..C.....G...........A...A.....G.......GG....C..C
P. alecto_A7(2)           ...C.....TG.....A.C..G..C......T....G...G.CA.A.....C..C.....G...........A...A.....G.......GG....C..C
H. sapiens_A7             ....G....T......A....T.....T...T.T..G...........A..C....................G...A....TG....AG.G........C
H. sapiens_A7A            ....G....T......A....T.....T...T.T..G...........A..C......G.............G...A....TG.....G.G.........
P. troglodytes_A7         ....G....T.G....A....T.....T...T.T..G...........A..C....................G...A....TG....AG..........C
P. troglodytes_A7A        ....G....T....G.A....T.....T...T.T..A...........A..C....................G...A....TG.....G.G.........
P. paniscus_A7            ....G....T.G....A....T.....T...T.T..G...........A..C....................G...A....TG....AG..........C
P. paniscus_A7A           ....G....T....G.A....T.....T...T.T..G..............C....................G...A....TG.....G.G.........
G. gorilla_A7             ....G....T......A....T.....T...T.T..G...........A..C....................G...A....TG.....G.G..A.....C
G. gorilla_A7A            ....G....T......A....T.....T...T.T..G...........A..C...................GG...A....TG.....G.G.........
N. leucogenys_A7          ....G....T......A....T.....T...T.T..G...........A..C....................A...A.....G.....G.G........C
N. leucogenys_A7A         ....G....T......A....T.....T...T.T..G...........A..C.................T..G...A.....G.....G.G........C
P. anubis_A7A             ....G....T......A....T.....T...T.T..G...........A..C....................G...A.....G...G.G..........C
M. mulatta_A7             ....G....T......A....T.....T...T.T..G...........A..C....................G..AA.....GA..G.G.........TC
M. fascicularis_A7A       ....G....T......A....T.....T...T.T..G...........A..C....................G..AA.....GA..G.G.........TC
M. fascicularis_A7        ....G....T......A....T.....T...T.T..G...........A..C....................G..AA.....GA..G.G.........TC
M. nemestina_A7A          .........T......A....T.....T...T.T..G...........A..C....................G..AA.....G...G.G.G........C
R. bieti_A7A              ....G....T......A....T.....T...T.T..G...........A..C....................G...A.....G...G.GAG..A.....C
R. bieti_A7               ....G....T......A....T.....T...T.T..G...........A..C....................G...A.....G...G.GAG..A.....C
C. capucinus_A7(1)        ....GG...T......A....T.....T...TAT..G...C.T.....A..C.......TT...........G...A.....G.....GAG........C
S. boliviensis_A7(1)      ....GG...T......A....T.....T...TAT..G...C.T.....A..C.......TT...........G...A.....G....AGAG........C
O. afer_A7(1)             ....C.T..T......A....G.....T...T.T..G...A.T.....A..A.....T..............A...A....TG.T..AGA..........
O. afer_A7(2)             ...CC.T..T......A....G.....T...T.T..G...A.T.....A..A.....T..............A...A....TG.T..AGA..........
L. africana_A7(1)         .........T......A..............T.T.....TG.AG....A..C.......AT..T........A...A....TG.T.....G........C
```

```
                             310
                        ....|....|....|
B. acutorostrata_A7(1)  GGGGGAAAT---TCG
B. tauros_A7(1)         .........---CA.
B. tauros_A7(2)         .........---CA.
B. tauros_A7(3)         .........---CA.
B. indicus_A7(1)        .........---CA.
B. indicus_A7(2)        .........---CA.
B. indicus_A7(3)        .........---CA.
B. bison_A7(1)          .........---CA.
B. bison_A7(1)          .........---CA.
B. bubalis_A7(1)        .........---CA.
B. bubalis_A7(2)        .........---AA.
B. bubalis_A7(3)        .........---CA.
P. hodgsonii_A7(1)      .........---CA.
P. hodgsonii_A7(2)      .........---CA.
C. hircus_A7(1)         .........---AA.
C. hircus_A7(2)         .........---CA.
C. hircus_A7(3)         .........---AA.
O. aries_A7(1)          .........---CA.
O. aries_A7(2)          .........---AA.
C. dromedarius_A7(1)    .....CG..---CA.
C. ferus_A7(1)          .....CG..---CA.
C. bactrianus_A7(1)     .....CG..---CA.
V. pacos_A7(1)          .....C...---CA.
S. scrofa_A7(1)         CC....C..---CA.
S. scrofa_A7(2)         CC..A.C..---CA.
A. melanoleuca_A7(1)    .T....GGC---CA.
U. arctos_A7(1)         AC.....GC---CA.
U. maritimus_A7(1)      AC.....GC---CA.
C. ursinus_A7(1)        .......GC---.G.
E. asinus_A7(1)         .AA...G.C---CA.
E. caballus_A7(1)       .AA...G.C---CA.
M. lucifugus_A7(1)      ..T...GGA---CAC
M. lucifugus_A7(2)      ......GGA---CAC
M. lucifugus_A7(3)      ......GGA---CAC
M. lucifugus_A7(4)      ......GGA---CAC
M. lucifugus_A7(5)      ......GGA---CAC
M. lucifugus_A7(6)      ......GGA---CAC
M. lucifugus_A7(7)      ......GGA---CAC
M. lucifugus_A7(8)      ..T..GGGAGGACAA
M. lucifugus_A7(9)      ......GGA---CAC
M. lucifugus_A7(10)     ......GGA---CAC
M. lucifugus_A7(11)     ......GGA---CAC
M. lucifugus_A7(12)     ......GGA---CAC
M. lucifugus_A7(13)     .......GA---CA.
M. brandtii_A7(4)       ......GGA---CA.
M. brandtii_A7(1)       ..T...GGA---CAC
M. brandtii_A7(5)       .......GA---CA.
M. brandtii_A7(2)       ......GGA---CAC
M. davidii_A7(2)        ..T...GGA---CAC
M. davidii_A7(1)        .......GA---GA.
M. davidii_A7(3)        ......GGA---CAC
M. natalensis_A7(1)     .......GG---CA.
M. natalensis_A7(2)     .......GG---CA.
E. fuscus_A7(1)         .......GA---CA.
R. aegyptiacus_A7(1)    .....GCGC---CA.
R. aegyptiacus_A7(3)    .....GCGC---CA.
R. aegyptiacus_A7(2)    .....GCGC---CA.
P. vampyrus_A7(1)       .....G.GC---CA.
P. alecto_A7(1)         .....G.GC---CA.
P. alecto_A7(2)         .....G.GC---CA.
H. sapiens_A7           .....C.GC---CA.
H. sapiens_A7A          .......GC---CA.
P. troglodytes_A7       ....A..GC---CA.
P. troglodytes_A7A      .......GC---CA.
P. paniscus_A7          ....A..GC---CA.
P. paniscus_A7A         .......GC---CA.
G. gorilla_A7           .......GC---CA.
G. gorilla_A7A          .......GC---CA.
N. leucogenys_A7        .......GC---CA.
N. leucogenys_A7A       .......GC---CA.
P. anubis_A7A           ..A....GC---CA.
M. mulatta_A7           A......GC---CA.
M. fascicularis_A7A     A......GC---CA.
M. fascicularis_A7      A......GC---CA.
M. nemestina_A7A        .......GC---CA.
R. bieti_A7A            .......GC---CA.
R. bieti_A7             .......GC---CA.
C. capucinus_A7(1)      ...A...GC---CAC
S. boliviensis_A7(1)    .......GC---CA.
O. afer_A7(1)           ....A..GC---CA.
O. afer_A7(2)           ....A..GC---CA.
L. africana_A7(1)       ......TGC---CA.
```
