# Supplementary material for: The evolution of S100A7: an unusual gene expansion in Myotis bats
Source: BMC Evol Biol. 2019 May 14;19:102. doi: 10.1186/s12862-019-1433-0 (PMC6518696; doi:10.1186/s12862-019-1433-0)
Supplement: Supplementary file 4 — Alignment of S100A15 proteins from several eutherian mammals. Dots = identity with Mus musculus S10015 protein. (PDF 172 kb) [file 12862_2019_1433_MOESM4_ESM.pdf]

Additional File 4. Alignment of S100A15 proteins from several eutherian mammals. Dots = identity with *Mus musculus* S10015 protein.

```
                                     10        20        30        40        50        60        70        80        90       100
                             ....|....|....|....|....|....|....|....|....|....|....|....|....|....|....|....|....|....|....|....|
Mus musculus                 MPDTPVEDSLFQIIHCFHHYAAREGDKETLSLEELKALLLDSVPRFMDTLGRRQPYYITELFRAADKNKDNQICFDEFLYILGKLVKDYHLQFHRQLCAH XM_006501635.3
Mus musculus                 .................................................................................................... AY582964.1
Mus musculus                 .................................................................................................... NM_199422.1
Mus caroli                   ...............................................................V.................................... XM_021159181.1
Mus pahari                   .................................................................................................... XM_021197148.1
Rattus norvegicus            ..................................................................................................T. NM_001109471.1
Ochotona princeps            ..E..M.E........Y.Q.......V.........D..M.N.....EA...KE....A...................E....V...........Y....... XM_012928175.1
Oryctolagus cuniculus        .T...M........R...Q.......V.....D......M.NA....EG...K..C.V........R...............V....A......Y..R.... XM_002715514.3
Tupaia chinensis             .T.....E........Y.Q......NA.............M.N.....ES...KE....A.................................Y....... XM_006159835.1
Pan troglodytes              .T.....E..........Q....Q..M.....Q..Q...M.N......S...KE..........Q..............................Y.W..... XM_003949560.4
Pan paniscus                 .T.....E..........Q...WQ..M.....Q..Q...M.N......S...KE..........Q..............................Y....... XM_003817182.1
Gorilla gorilla              .T.....E..........Q....Q..M.....Q..Q...M.NM.....S.L..E...V....Q.T...R...........................Y......R XM_004026728.1
Pongo abelii                 .T.....E..........Q....Q..M.....Q..Q...M.NM.....S...KE..........Q..............................Y......R NM_001135536.1
Nomascus leucogeny           .T.....E..........Q....Q..M.....Q..Q...M.N......S...KE.....D..Q..............................Y......R XM_004089898.1
Capra hircus                 .T.....E........Y.E.......V..............T.N.....E....KE.C...Q........Q......E..............W.Y....... XM_005677512.3
Bison bison bison            .T.....E........Y.E.......A..............M.N.....E....KE.....Q........Q......E................Y....... XM_010857539.1
Bos taurus                   .T.....E........Y.E.......A..............M.N.....E....KE.....Q........Q......E................Y....... XM_002686003.5
Bos taurus                   .T.....E........Y.E.......A..............M.N.....E....KE.....Q........Q......E................Y....... XM_010802984.3
Bos indicus                  .T.....E........Y.E.......A..............M.N..X..E....KE.....Q........Q......E................Y....... XM_019958160.1
Bos mutus                    .T..R..E........Y.E.......A..............M.N.....E....KE.....Q........Q......E................Y....... XM_005909578.1
Odocoileus virginianus       .T.....E........Y.E.......V..............T.N.....E....KE.....Q..Q.....R......E................Y....... XM_020907557.1
Bubalus bubalis              .T.....E........Y.E.......V..............M.N.....E....KE.....Q........Q......E................Y....... XM_006051695.2
Equus asinus                 .T...A.E........Y.Q.......V..............M.N.....ES...KE................................L......Y.Q..... XM_014864980.1
Equus przewalskii            .T...A.E........Y.Q.......V..............M.N.....ES...KE.H..............................L......Y.Q..... XM_008543015.1
Equus caballus               .T...A.E........Y.Q.......V..............M.N.....ES...KE.H..S...........................L......Y.Q..... XM_005610244.3
Panthera pardus              .TG....E........Y.Q.......V..............M.N.....ES...KE....S....................FV..R..R....HY...Q... XM_019431268.1
Panthera tigris              .TG....E........Y.Q.......V..............M.N.....ES...KE....S....................FV..R..R....HY...Q... XM_015540143.1
Puma concolor                .TG....E........Y.Q.......V..............M.N.....ES...KE....S.......R............FV..R..R....HY...Q... XM_025915301.1
Felis catus                  .TG....E........Y.Q.......V..............M.NA....ES...KE....S....................FV..R..R....HY...Q... XM_019821408.2
Acinonyx jubatus             .TG....E........Y.Q.......V..............M.N.....ES...KE....S....................FV..R..R....HY...Q... XM_015085468.1
Desmodus rotundus            .R..A..E.......SY.Q.......V..............M.N......S...KE..F..........D...............R.LR.....Y....... XM_024570051.1
Desmodus rotundus            .R..A..E.......SY.Q.......V..............M.N......S...KE..F..........D...............R.LR.....Y....... XM_024570759.1
Myotis lucifugus             .T.....E.......SY.Q.......V..............R.N.....ES...EK..F..........D............V..Q.LR.....Y....... XM_023759213.1
Myotis lucifugus             .T.....E.......SY.Q.......V..............R.N.....ES...EK..FV.........D............V..Q.LR.....Y....... XM_014461372.2
Myotis brandtii              .T.....E.......SY.Q.......V..............M.N.....ES...EK..F..........D............V..Q.LR.....Y....... XM_014540493.1
Myotis brandtii              .T.....E.......SY.Q.......V..............M.N.....ES...EK..F..........D............V..R.LR.....Y....... XM_014540726.1
Rhinolophus sinicus          .T.....E....L...Y.Q.......V..............M.T.....ES...KE..F.A...............E.........R.LR.....Y....... XM_019713233.1
Hipposideros armiger         TM.....E....L...Y.Q.......V..............M.N.....ES...KE..F.A........D....L.......V..R.LR.....Y....... XM_019627360.1
Pteropus alecto              .T.....E..V.LVR.Y.R.......V.......Q......E.NT...LGS....E..F.S........D....LS......V..R.LR.C...Y..R.... XM_006923669.1
Pteropus vampyrus            .T.....E..V.LVR.Y.R.......V.......R..R...E.NA...LGS....E..F.S........D....LS......V..R.LR.C...Y....... XM_023523867.1
Rousettus aegyptiacus        .T.....E..V.LVR.Y.Q.......V.......Q......E.N....L.S....E..F.S........D....LS......V....LR.C...Y..R...Q XM_016126056.1
Canis lupus                  .THK.M.E...V..Y.Q.......V..............M.N..C..ES...KE....S....................F...R.L.....LY......C XM_014115423.2
```

```
                             ....|....
Mus musculus                 YCTEHSLY* XM_006501635.3
Mus musculus                 ........* AY582964.1
Mus musculus                 ........* NM_199422.1
Mus caroli                   ........* XM_021159181.1
Mus pahari                   ...Q....* XM_021197148.1
Rattus norvegicus            ...Q.N..* NM_001109471.1
Ochotona princeps            H.AQ.N..* XM_012928175.1
Oryctolagus cuniculus        H.AQ....* XM_002715514.3
Tupaia chinensis             .SAQ.N..* XM_006159835.1
Pan troglodytes              ..AQ....* XM_003949560.4
Pan paniscus                 ..AQ....* XM_003817182.1
Gorilla gorilla              ..AQ....* XM_004026728.1
Pongo abelii                 ..AQ....* NM_001135536.1
Nomascus leucogeny           ..AQ....* XM_004089898.1
Capra hircus                 H.SQ....* XM_005677512.3
Bison bison bison            ...Q....* XM_010857539.1
Bos taurus                   ...Q....* XM_002686003.5
Bos taurus                   ...Q....* XM_010802984.3
Bos indicus                  ...Q....* XM_019958160.1
Bos mutus                    ...Q....* XM_005909578.1
Odocoileus virginianus       ...Q....* XM_020907557.1
Bubalus bubalis              ...Q....* XM_006051695.2
Equus asinus                 ..SQ....* XM_014864980.1
Equus przewalskii            ..SQ....* XM_008543015.1
Equus caballus               ..SQ....* XM_005610244.3
Panthera pardus              ..AQ....* XM_019431268.1
Panthera tigris              ..AQ....* XM_015540143.1
Puma concolor                ..AQ...H* XM_025915301.1
Felis catus                  ..AQ...H* XM_019821408.2
Acinonyx jubatus             ..AQ...H* XM_015085468.1
Desmodus rotundus            ...Q....* XM_024570051.1
Desmodus rotundus            ...Q....* XM_024570759.1
Myotis lucifugus             ..AQ....* XM_023759213.1
Myotis lucifugus             ..AQ....* XM_014461372.2
Myotis brandtii              ..AR....* XM_014540493.1
Myotis brandtii              ..AR....* XM_014540726.1
Rhinolophus sinicus          ..AQ....* XM_019713233.1
Hipposideros armiger         ..AQ....* XM_019627360.1
Pteropus alecto              C.AQR...* XM_006923669.1
Pteropus vampyrus            R.AQR...* XM_023523867.1
Rousettus aegyptiacus        R.AQRG..* XM_016126056.1
Canis lupus                  ..AR...H* XM_014115423.2
```
