# Supplementary material for: The evolution of S100A7: an unusual gene expansion in Myotis bats
Source: BMC Evol Biol. 2019 May 14;19:102. doi: 10.1186/s12862-019-1433-0 (PMC6518696; doi:10.1186/s12862-019-1433-0)

Additional File 5. Search for the gene loss remnants of *S100A7* and *S100A15* from mouse and human, respectively.

Figure 1. Alignment of a partial sequences of *S100A7* from mouse. The abbreviations correspond to the following species: H. sapiens - *Homo sapiens*; M. musculus - *Mus musculus*. Dots = identity with *H. sapiens S100A7* sequence.

```
                                  10        20        30        40        50        60        70        80        90       100
                          ....|....|....|....|....|....|....|....|....|....|....|....|....|....|....|....|....|....|....|....|
H. sapiens-S100A7         ATGAGCAACACTCAAGCTGAGAGGTCCATAATAGGCATGATCGACATGTTTCACAAATACACCAGACGTGATGACAAGATTGAGAAGCCAAGCCTGCTGA
M. musculus-Pseudo_S100A7 ...-A..G...CA.......A.A......GG.G...GGA...A..T.......TG...C..GGGATAA...CA...T...CA........GATGG.AA..

                                 110       120       130       140       150       160       170       180       190       200
                          ....|....|....|....|....|....|....|....|....|....|....|....|....|....|....|....|....|....|....|....|
H. sapiens-S100A7         CGATGATGAAGGAGAACTTCCCCAACTTCCTTAGTGCCTGTGACAAAAAGGGCACAAATTACCTCGCCGATGTCTTTGAGAAAAAGGACAAGAATGAGGA
M. musculus-Pseudo_S100A7 A...AT..........T....A.........C.AA.T.....TG..T.GAA.T.T..C..TT.AGTATTTGT.GGGCTCTGCTGT.A.ACTC.GA.GC.T

                                 210       220       230       240       250       260       270       280       290       300
                          ....|....|....|....|....|....|....|....|....|....|....|....|....|....|....|....|....|....|....|....|
H. sapiens-S100A7         TAAGAAGATTGATTTTTCTGAGTTTCTGTCCTTGCTGGGAGACATAGCCACAGACTACCACAAGCAGAGCCATGGAGCAGCGCCCTGTTCCGGGGGCAGC
M. musculus-Pseudo_S100A7 CT..TTT.ACCC.A.GCTGA.AAGGT

                          ...
H. sapiens-S100A7         CAG
M. musculus-Pseudo_S100A7
```

Figure 2. Genomic location of the partial *S100A7* sequence from mouse. Data was obtained using *S100A7* coding sequence from human as a reference against Ensembl database.

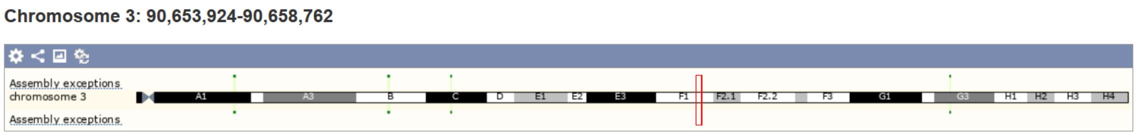

Figure 3. Alignment of a partial sequences of *S100A15* from human. The abbreviations correspond to the following species: M. musculus - *Mus musculus;* P. troglodytes - Pan troglodytes; H. sapiens - *Homo sapiens*. Dots = identity with *M. musculus S100A15* sequence.

```
                                  10        20        30        40        50        60        70        80        90       100
                          ....|....|....|....|....|....|....|....|....|....|....|....|....|....|....|....|....|....|....|....|
M. musculus-S100A15       ATGCCAGACACACCAGTGGAGGACTCCCTCTTCCAAATCATACACTGCTTCCATCACTATGCTGCCCGGGAAGGGGACAAGGAGACCTTGTCCCTGGAGG
P. troglodytes-S100A15    ...A.......C...........A.................C...........C..G.....A......C....T..T.T.......C........C...
H. sapiens-Pseudo_S100A15 ----------------------------------------------------------------------------------------------------

                                 110       120       130       140       150       160       170       180       190       200
                          ....|....|....|....|....|....|....|....|....|....|....|....|....|....|....|....|....|....|....|....|
M. musculus-S100A15       AGTTGAAAGCTCTGCTCTTGGATAGTGTGCCTCGCTTCATGGACACCTTGGGCCGCAGGCAGCCATACTACATCACAGAGCTGTTCCGGGCAGCTGACAA
P. troglodytes-S100A15    .....C.G..C......A....C.AC.....C.............G.........G.A.G..........T.........T......A...G..A.....
H. sapiens-Pseudo_S100A15 ----------------------------------------------TCA......G.A.G..........T.........T......A...C..A.....

                                 210       220       230       240       250       260       270       280       290       300
                          ....|....|....|....|....|....|....|....|....|....|....|....|....|....|....|....|....|....|....|....|
M. musculus-S100A15       AAACAAGGACAACCAGATCTGCTTTGATGAGTTCCTGTACATCTTGGGCAAGCTGGTGAAGGACTACCATCTCCAGTTCCACCGGCAGTTGTGTGCACAC
P. troglodytes-S100A15    G.......................C..C........C................................C..G....A....T.....C.......C...
H. sapiens-Pseudo_S100A15 G.......................C...........C................................C..G..C.A.....A....C.......C.G.

                                 310       320
                          ....|....|....|....|....|..
M. musculus-S100A15       TACTGTACTGAGCACAGCCTCTACTAG
P. troglodytes-S100A15    ......G.CC.A...............
H. sapiens-Pseudo_S100A15 ......G.CC.A...............
```

Figure 4. Genomic location of the partial *S100A15* sequence from human. Data was obtained using *S100A15* coding sequence from mouse as a reference against Ensembl database.

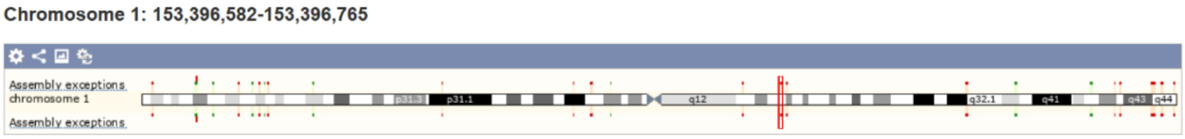

Supplement: Supplementary file 5 — Search for the gene loss remnants of S100A7 and S100A15 from mouse and human, respectively. (PDF 193 kb) [file 12862_2019_1433_MOESM5_ESM.pdf]
