# Supplementary material for: The evolution of S100A7: an unusual gene expansion in Myotis bats
Source: BMC Evol Biol. 2019 May 14;19:102. doi: 10.1186/s12862-019-1433-0 (PMC6518696; doi:10.1186/s12862-019-1433-0)
Supplement: Supplementary file 6 — Alignment of partial sequences S100A7 from bats. The abbreviations correspond to the following species: M. lyra - Megaderma lyra; R. ferrumequinum - Rhinolopus ferrumequinum; R. sinicus - Rhinolophus sinicus; P. patnellii - Pteronotus parnellii; H. arimiger - Hipposideros armiger; D. rotundus - Desmodus rotundus. Dots = identity with M. lucifugus_A7(1) sequence. (PDF 149 kb) [file 12862_2019_1433_MOESM6_ESM.pdf]

Additional file 6. Alignment of partial sequences *S100A7* from bats. The abbreviations correspond to the following species: *M. lyra - Megaderma lyra*; *R. ferrumequinum - Rhinolopus ferrumequinum*; *R. sinicus - Rhinolophus sinicus*; *P. patnellii - Pteronotus parnellii*; *H. arimiger - Hipposideros armiger*; *D. rotundus - Desmodus rotundus*. Dots = identity with *M. lucifugus*_A7(1) sequence.

```
                                       10        20        30        40        50        60        70        80        90       100
                               ....|....|....|....|....|....|....|....|....|....|....|....|....|....|....|....|....|....|....|....|
M. lucifugus_A7(1)             ATGAGTCAAACTCAAGTTGAGAAGTCCATGATGGACTTGGTTGTCCTGTTCCACAAATACACCAAACCCGATGACAAGATTGACAAGCGGGGCCTGCTGA
M. lyra scaffold_104088        --------------------------------------------.....C..........CT..G....AG.....G..CA..TG.T.C........CA.
M. lyra scaffold_28774         ----------------------------------------------------------------------------------------------------
R. ferrumequinum_unplaced scaf ----------------------------------------------------------------------------------------------------
R. ferrumequinum_unplaced scaf ----------------------...G.G...........A...A.T....T...........G.GC..........C.G..A.T...GA........A.G
R sinicus1 scaffold_74394      ----------------------...G.G...........A...A.T....T...........G.GC..........C.G..A.T...GA........A.G
R. sinicus2_scaffold_74394     ----------------------------------------------------------------------------------------------------
P. parnellii_scaffold_8346     .....C..C...GC..C..........G......G.A..A.C.A...C............GT...G..........C...C.......C...........
P. parnellii_scaffold_27792    ----------------------------------------------------------------------------------------------------
H. armiger_unplaced scaffold   -------------------------------------..A...A.....C.......C...T..G...------...---........T........C..
H. armiger_unplaced scaffold   ----------------------------------------------------------------------------------------------------
D. rotundus_unplaced scaffold  .....C..C...G...C..........G......G.A..A.C.A................GT..............CA......G...C.........A.
D. rotundus_unplaced scaffold  ----------------------------------------------------------------------------------------------------

                                      110       120       130       140       150       160       170       180       190       200
                               ....|....|....|....|....|....|....|....|....|....|....|....|....|....|....|....|....|....|....|....|
M. lucifugus_A7(1)             AGATGCTGAGGGAGAATTTCCCTATATTCCTCAAAGCCTGTGACAAAAAGGGCAATGATTTCTTGGACCATATCTTTGAGGAAAAGGACAAGAATAAGGA
M. lyra scaffold_104088        ...G....---...........C.AC........G.............-...T..A.G..A....TC..C.................C.......G....
M. lyra scaffold_28774         ---------------------------------------------...G....GTA....A....TC...C.....C.....C............CG...
R. ferrumequinum_unplaced scaf -----------------------------------------.......T......G....A....TCAGG.G..........C..C.........C.C..
R. ferrumequinum_unplaced scaf .........A....T.......ACAC.......GG.......----------------------------------------------------------
R sinicus1 scaffold_74394      .........A....T.......ACAC.......GG.......----------------------------------------------------------
R. sinicus2_scaffold_74394     -----------------------------------------.......T......G....A....TCAGG.G..........C..C.........C.C..
P. parnellii_scaffold_8346     C........A......C.....C.AC......G.G..---------------------------------------------------------------
P. parnellii_scaffold_27792    --------------------------------------------------------....A....TC...............C..A.........GG...
H. armiger_unplaced scaffold   ..G......A............C.AC...........T.AC.......G......-A..AA....TC...........C........C.........-..
H. armiger_unplaced scaffold   -----------------------------------------..AC...T......A....A....TC.A...........A.G.........G..C....
D. rotundus_unplaced scaffold  .........A............C.AC......G.G..---------------------------------------------------------------
D. rotundus_unplaced scaffold  -----------------------------------------.....T........A....A....TC..G.........A.....A..............

                                      210       220       230       240       250       260       270       280       290       300
                               ....|....|....|....|....|....|....|....|....|....|....|....|....|....|....|....|....|....|....|....|
M. lucifugus_A7(1)             TAAGAAGATCGAGTTTTCCGAGTTTCTGTCGGTGTTGGGAGTCATAGCCACGGACTACCACAATCAGAGCCACGGAACCCCGCTCTGTTCCGGTGGAGGA
M. lyra scaffold_104088        .......C.TTG...........G......CT..G....G.A....C....A....-G...C..T.......T...G.----------------------
M. lyra scaffold_28774         .C.......TC.......T.....C.....CT.......G.A...C.....C...........C............G.GG...C......T..G...---
R. ferrumequinum_unplaced scaf .........TC....C..............C..AC....GAG...T.....A..T...........C.........G.GGAA......G.T..G.-----
R. ferrumequinum_unplaced scaf ----------------------------------------------------------------------------------------------------
R sinicus1 scaffold_74394      ----------------------------------------------------------------------------------------------------
R. sinicus2_scaffold_74394     .........TC....C..............C..AC....GAG...T.....A..T...........C.........G.GGAA......G.T..G.-----
P. parnellii_scaffold_8346     ----------------------------------------------------------------------------------------------------
P. parnellii_scaffold_27792    .........TC.......................G....G.A...C...........................AA.G.GG.CGC.A....T..G...---
H. armiger_unplaced scaffold   .........TC.......T.........C.CT..G....G.A....T....A.............T......T...G-----------------------
H. armiger_unplaced scaffold   .........T..T.................CT.AG....G.C...T..................T.T.C.......G.AGA............G.-----
D. rotundus_unplaced scaffold  ----------------------------------------------------------------------------------------------------
D. rotundus_unplaced scaffold  .........TC.......T...........CT..G....G.A.........A..A..................AA.GTGA.CGC.A....T..G.-----

                               ...
M. lucifugus_A7(1)             CAC
M. lyra scaffold_104088        ---
M. lyra scaffold_28774         ---
R. ferrumequinum_unplaced scaf ---
R. ferrumequinum_unplaced scaf ---
R sinicus1 scaffold_74394      ---
R. sinicus2_scaffold_74394     ---
P. parnellii_scaffold_8346     ---
P. parnellii_scaffold_27792    ---
H. armiger_unplaced scaffold   ---
H. armiger_unplaced scaffold   ---
D. rotundus_unplaced scaffold  ---
D. rotundus_unplaced scaffold  ---
```
