# Supplementary material for: The evolution of S100A7: an unusual gene expansion in Myotis bats
Source: BMC Evol Biol. 2019 May 14;19:102. doi: 10.1186/s12862-019-1433-0 (PMC6518696; doi:10.1186/s12862-019-1433-0)

Additional File 7. Selection (dN/dS) analyses

Table 1. Branch model results obtained in PAML for the *M. lucifugus*_A7(8) and *M. brandtii*_A7(4) branch.

Model	lnL	2ΔlnL	p-value	ω (dN/dS)
One ratio	-4371.805			
Two-rates	-4367.419	8.77	< 0.005	1.44

[1] P-value < 0.05 is used to reject the null hypothesis of equal rates between lineages

Table 2. Test for Selection Relaxation on the *M. lucifugus*_A7(8) and *M. brandtii*_A7(4) branch. The *M. lucifugus*_A7(8) and *M. brandtii*_A7(4) branch was the "test" branch, and all the primates were assigned as "reference" branches.

Model	ω_1	ω_2	ω_3	*log* L	*P*	AICc	Branch set
General descriptive	0.00 (57.53%)	0.97 (37.36%)	8.15 (5.11%)	-3112.9	137	6512.3	Shared
RELAX alternative	0.00 (28.59%)	1.00 (64.84%)	291.70 (6.57%)	-3143.9	84	6460.5	Test
	0.00 (28.59%)	1.00 (64.84%)	1.12 (6.57%)				Reference
RELAX null	0.00 (0.86%)	0.36 (81.15%)	2.85 (17.98%)	-3146.6	83	6463.7	Test
	0.00 (0.86%)	0.36 (81.15%)	2.85 (17.98%)				Reference
RELAX partitioned descriptive	1.00 (14.36%)	1.00 (78.85%)	5001.80 (6.79%)	-3140.5	88	6462.1	Test
	0.27 (100.00%)	0.27 (0.00%)	1.00 (0.00%)				Reference

Note. Log Likelihood Values and Parameter Estimates for the RELAX analysis. ω1, first omega rate class; ω2, second omega rate class; ω3, third omega rate class; p, number of parameters in the model; lnL, log likelihood value under the model; AICc, small-sample correct Akaike Information Criterion; Branch set, indicates which branch set each. Test for selection intensification (K = 50.00) was significant (P = 0.031, LR = 4.64).

Figure 1. ω distributions under the RELAX alternative model. The *M. lucifugus*_A7(8) and *M. brandtii*_A7(4) branch was the "test" branch, and all the primates were assigned as "reference" branches. Test branch is shown in green and reference branches are shown in black. For the RELAX test, we reduced the dataset from 81 to 36 sequences to reduce running time.

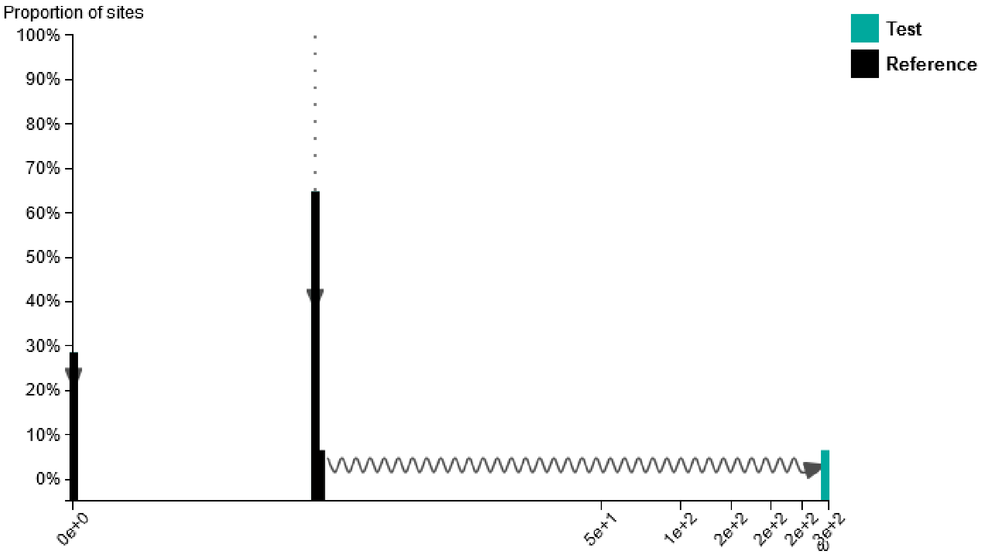

Supplement: Supplementary file 7 — Selection (dN/dS) analyses. (PDF 331 kb) [file 12862_2019_1433_MOESM7_ESM.pdf]
