# Supplementary material for: The evolution of S100A7: an unusual gene expansion in Myotis bats
Source: BMC Evol Biol. 2019 May 14;19:102. doi: 10.1186/s12862-019-1433-0 (PMC6518696; doi:10.1186/s12862-019-1433-0)
Supplement: Supplementary file 3 — Alignment between S100A15 and S100A7 proteins. Alignment between two S100A15 proteins from mouse, one S100A15 from P. troglodytes and two S100A7 proteins from H. sapiens and P. paniscus. Dots = identity with M. musculus_S100A7_XM_ 006501635.3 protein. (PDF 305 kb) [file 12862_2019_1433_MOESM3_ESM.pdf]

Additional File 3. Alignment between two S100A15 proteins from mouse, one S100A15 from *P. troglodytes* and two S100A7 proteins from *H. sapiens* and *P. paniscus*. Dots = identity with *M. musculus*_S100A7_XM_006501635.3 protein.

```
                                   10        20        30        40        50        60        70        80        90       100
                          ....|....|....|....|....|....|....|....|....|....|....|....|....|....|....|....|....|....|....|....|
M. musculus_S100A7A       MPDTPVEDSLFQIIHCFHHYAAREGDKETLSLEELKALLLDSVPRFMDTLGRRQPYYITELFRAADKNKDNQICFDEFLYILGKLVKDYHLQFHRQLCAH  XM_006501635.3
M. musculus_S100A7        ....................................................................................................  AY582964.1
P. troglodytes_S100A15    .T.....E..........Q....Q..M.....Q..Q...M.N......S...KE........Q.............................Y.W.....  XM_003949560.4
H. sapiens_S100A7         .SN.QA.R.IIGM.DM..K.TR.DD---KIEKPS.LTMMKENF.N.LSACDKKGTN.LADV.EKK...E.KK.D.S...SL..DIAT...K.S.GAAPCS  ENST00000368723
P. paniscus_S100A7        .SN.QA.R.IIGM.DM..K.TR.DD---KIDKPS.LTMMKENF.N.LSACDKKGTN.LANV.EKK...E.RK.D.S...SL..DIAT...K.S.GAAPCS  ENSPPAG00000042606

                          ....|....
M. musculus_S100A7A       YCTEHSLY*  XM_006501635.3
M. musculus_S100A7        ........*  AY582964.1
P. troglodytes_S100A15    ..AQ....*  XM_003949560.4
H. sapiens_S100A7         GGSQ       ENST00000368723
P. paniscus_S100A7        GESQ       ENSPPAG00000042606
```
